# Human neutrophils can mimic myeloid-derived suppressor cells (PMN-MDSC) and suppress microbead or lectin-induced T cell proliferation through artefactual mechanisms

**DOI:** 10.1038/s41598-018-21450-6

**Published:** 2018-02-16

**Authors:** Dmitri Negorev, Ulf H. Beier, Tianyi Zhang, Jon G. Quatromoni, Pratik Bhojnagarwala, Steven M. Albelda, Sunil Singhal, Evgeniy Eruslanov, Falk W. Lohoff, Matthew H. Levine, Joshua M. Diamond, Jason D. Christie, Wayne W. Hancock, Tatiana Akimova

**Affiliations:** 1 The Pathology Bioresource, Department of Pathology and Laboratory Medicine, University of Pennsylvania, Philadelphia, PA 19104 USA; 2 Division of Nephrology, Department of Pediatrics, Children’s Hospital of Philadelphia and University of Pennsylvania, PA 19104 Philadelphia, USA; 3 Division of Transplant Immunology, Department of Pathology and Laboratory Medicine, Children’s Hospital of Philadelphia and University of Pennsylvania, Philadelphia, PA USA; 4 Division of Thoracic Surgery, Department of Surgery, University of Pennsylvania, 19104 Philadelphia, PA USA; 5 Division of Pulmonary, Allergy and Critical Care Medicine, Department of Medicine, University of Pennsylvania, Philadelphia, PA USA; 6 Section on Clinical Genomics and Experimental Therapeutics, National Institute on Alcohol Abuse and Alcoholism (NIAAA), National Institutes of Health (NIH), Bethesda, MD 20892-154 USA; 7 Department of Surgery, Penn Transplant Institute, Hospital of the University of Pennsylvania and University of Pennsylvania, Philadelphia, PA 19104 USA; 8 Department of Biostatistics and Epidemiology, University of Pennsylvania, Philadelphia, PA 19104 USA

## Abstract

We report that human conventional CD15^+^ neutrophils can be isolated in the peripheral blood mononuclear cell (PBMC) layer during Ficoll gradient separation, and that they can impair T cell proliferation *in vitro* without concomitant neutrophil activation and killing. This effect was observed in a total of 92 patients with organ transplants, lung cancer or anxiety/depression, and in 18 healthy donors. Although such features are typically associated in the literature with the presence of certain myeloid-derived suppressor cell (PMN-MDSC) populations, we found that commercial centrifuge tubes that contained membranes or gels for PBMC isolation led to up to 70% PBMC contamination by CD15^+^ neutrophils, with subsequent suppressive effects in certain cellular assays. In particular, the suppressive activity of human MDSC should not be evaluated using lectin or microbead stimulation, whereas assays involving soluble or plate-bound antibodies or MLR are unaffected. We conclude that CD15^+^ neutrophil contamination, and associated effects on suppressor assays, can lead to significant artefacts in studies of human PMN-MDSC.

## Introduction

Characterization of immune cells with suppressive properties is of major interest in transplantation^1,2^, autoimmunity^3,4^ and tumor immunology^5–8^. One such population, myeloid-derived suppressor cells (MDSC), has garnered much attention, with many publications over the last decade documenting their phenotypic and functional characteristics. While MDSC are heterogeneous, they demonstrate the ability to suppress T cell proliferation and cytokine production^9^. Characterization of murine MDSC is well established, but there is far less consensus as to the phenotype of their human counterparts^10,11^, thereby affecting the ability to compare data from different laboratories. At present, the ‘gold standard’ for defining MDSC is to correlate their phenotypic evaluation with functional assays, which typically involves assessing their ability to inhibit T cell proliferation *in vitro*^12^.

*In vitro* suppression assays can involve different methods of T cell stimulation. Traditionally, mitogenic lectins, such as phytohemagglutinin (PHA) and concanavalin A (Con A) were used. More recently, the use of anti-CD3 or anti-CD3/28 monoclonal antibody (mAb)-coated microbeads were developed and has gained in popularity, given its greater physiologic relevance. Usually, all such forms of T cell stimulation are perceived by researchers as tools to study corresponding biological processes in T cells, with little or no attention paid to the effects of such stimulation on non-T cells. In the current study, we describe unexpected and previously unnoticed effects of lectins on conventional neutrophils, and novel interactions of neutrophils with microbeads. Both features lead to remarkable but artefactual suppression of T cell division, a result that could be erroneously interpreted as reflecting suppression by PMN-MDSC. The goal of this paper is to inform researchers studying human MDSC that CD15^+^ neutrophils may co-localize with cellular fractions expected to be enriched for MDSC and mediate artefactual suppression.

## Results

### Occurrence of a CD4^−^ suppressive cell subset

**Figure 1:**
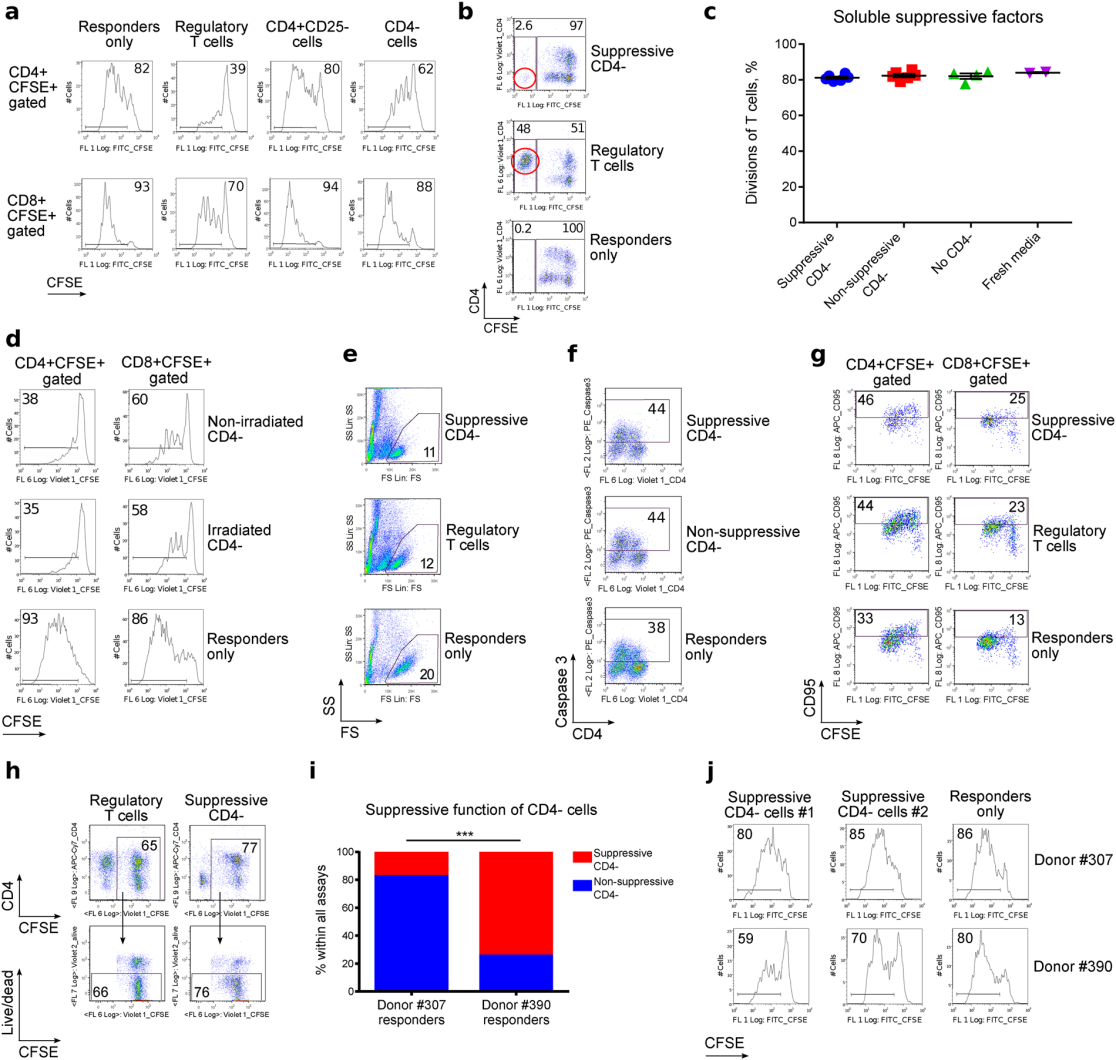
Suppressive human CD4^−^ cells. CFSE-labeled healthy donor PBMC were co-stimulated and incubated ± suppressive cells for 4 days. (**a**) Addition of Tregs and CD4^−^ PBMC impaired proliferation, whereas CD4^+^CD25^−^ T cells did not. Data representative of five independent experiments. (**b**) Live-gated suppressive cells (red circles) show that Treg, but not CD4^−^ cells, survive until the end of the experiment. In both examples, initial ratios of suppressive cells to responder cells were 1:1. Data representative of >40 experiments. (**c**) Media supernatant from suppression assays was mixed 1:1 with fresh media, and used in new suppression assays to test for soluble suppressive factors, which were not seen (2 experiments, 6 samples tested, p = 0.3063, Kruskal-Wallis test). (**d**) CD4^−^ cells retained suppressive properties independent of proliferation after γ-irradiation (2 experiments, 2 samples). (E–H) Responder PBMC did not undergo apoptosis or killing by suppressive CD4^−^ cells as shown by: FS/SS gating properties (**e**), Caspase 3 (**f**) CD95 expression (**g**), and live/dead staining (**h**). Data representative of >40 (**e**), 2 (**f**), 2 (**g**), and 2 (**h**) independent experiments with at least 2 samples in each one. (**i**,**j**) Comparison of different healthy donor responder cells. (**i**) Pooled data from 48 experiments (p < 0.0001, Fisher’s exact test). (**j**) Representative data showing donor responder PBMC cells exposed to CD4-depleted cells as suppressors. The CD4^−^ cells were isolated from anxiety/depression (#1) and adult kidney transplant (#2) patients. PBMC from Donor ID#390 were markedly easier to suppress. Presented histograms and dot plots consist of 4,826 ± 421 (mean ± SEM) events.

We have shown that human and murine FOXP3^+^ T-regulatory (Treg) cells divide vigorously during Treg suppression assays^13,14^. We questioned whether the effects of rapid cellular growth, with resultant deficiency of nutrients, were significant components of Treg suppressive function *in vitro*. To answer this, we stimulated and co-cultured peripheral blood mononuclear cells (PBMC) from healthy donors and transplant recipients, tracking the proliferation of each population by carboxyfluorescein succinimidyl ester (CFSE) labeling. We observed that PBMC of some patients had suppressive activity towards co-cultured allogeneic PBMC. To examine which cell subset was responsible for the suppressive effect, we isolated different PBMC fractions using CD4^+^CD25^+^ Treg isolation kits. As expected, Tregs exhibited suppressive capability (Fig. 1a). However, surprisingly, we also observed suppression by CD4-depleted PBMC (hereafter, CD4^−^). Unlike Tregs, very few of those cells were alive at the end of the suppression assay (Fig. 1b), though they had no signs of decreased viability at the beginning of the experiment.

To understand the mechanisms by which CD4^−^ cells impaired proliferation of responder T cells, we considered possible release of soluble suppressive factors into the media, CD4^−^ cell proliferation, and killing of the responder cells. However, cell culture media from CD4^−^ cells did not suppress T cell proliferation (Fig. 1c), and preventing CD4^−^ cell proliferation by prior γ-irradiation did not remove the suppressive effect on responder cells (Fig. 1d). Hence, neither cell overgrowth nor cell division appeared responsible. Furthermore, suppressive CD4^−^ cells did not generate increased proportions of dead or apoptotic responder cells, compared to Tregs with the same suppressive capabilities, or compared with non-suppressive CD4^−^ cells (Fig. 1e–h); i.e. they did not kill responder cells, but rather suppressed their proliferation.

In further experiments, we used a different source of healthy donor responder cells (changing from donor #307 to donor #390). This change led to even greater suppression by CD4^−^ suppressive cells (Table 1, Fig. 1i). Healthy donor PBMC that required higher levels of CD3 stimulation to reach equivalent responder cell divisions (i.e. cells from donor #390), were much more sensitive to suppression by CD4^−^ cells (Fig. 1i, j & Suppl. Fig. S1), suggesting that suppressive CD4^−^ cells affect the extent of TCR stimulation of responder cells. In summary, CD4^−^ cells from patients with different and unrelated diseases suppressed healthy donor T cell proliferation, and suppression did not involve (i) soluble factors, (ii) cell overgrowth, or (iii) killing of responder cells. We also found that suppression by CD4^−^ cells appeared to be related to limitation of TCR stimulation, and CD4^−^ suppressors were short-lived cells in comparison with lymphocytes.

**Table 1:** Occurrence of suppressive CD4^−^ cells in different clinical groups.

Type of clinical blood samples	Suppressive CD4^−^ cells observed, %	Suppressive CD4^−^ cells observed, #	Type of responder cells, donor ID#	Day of PBMC isolation
Pediatric kidney allograft	25	2 of 8	307	First
Pediatric liver allografts	14.3	3 of 21	307	First
Adult liver allografts	14.3	1 of 7	307	First
Adult kidney allografts	14.3	1 of 7	307	First
Adult kidney allografts, modified design*	100	4 of 4	390	First
Adult lung allograft	87.5	21 of 24	390	Second
Anxiety/depression study	92	11 of 12	390	Second

^*^For these experiments, responders from Donor #390 were used instead of #307.

### Identification of CD4^−^ suppressive cells and conditions for their isolation

**Figure 2:**
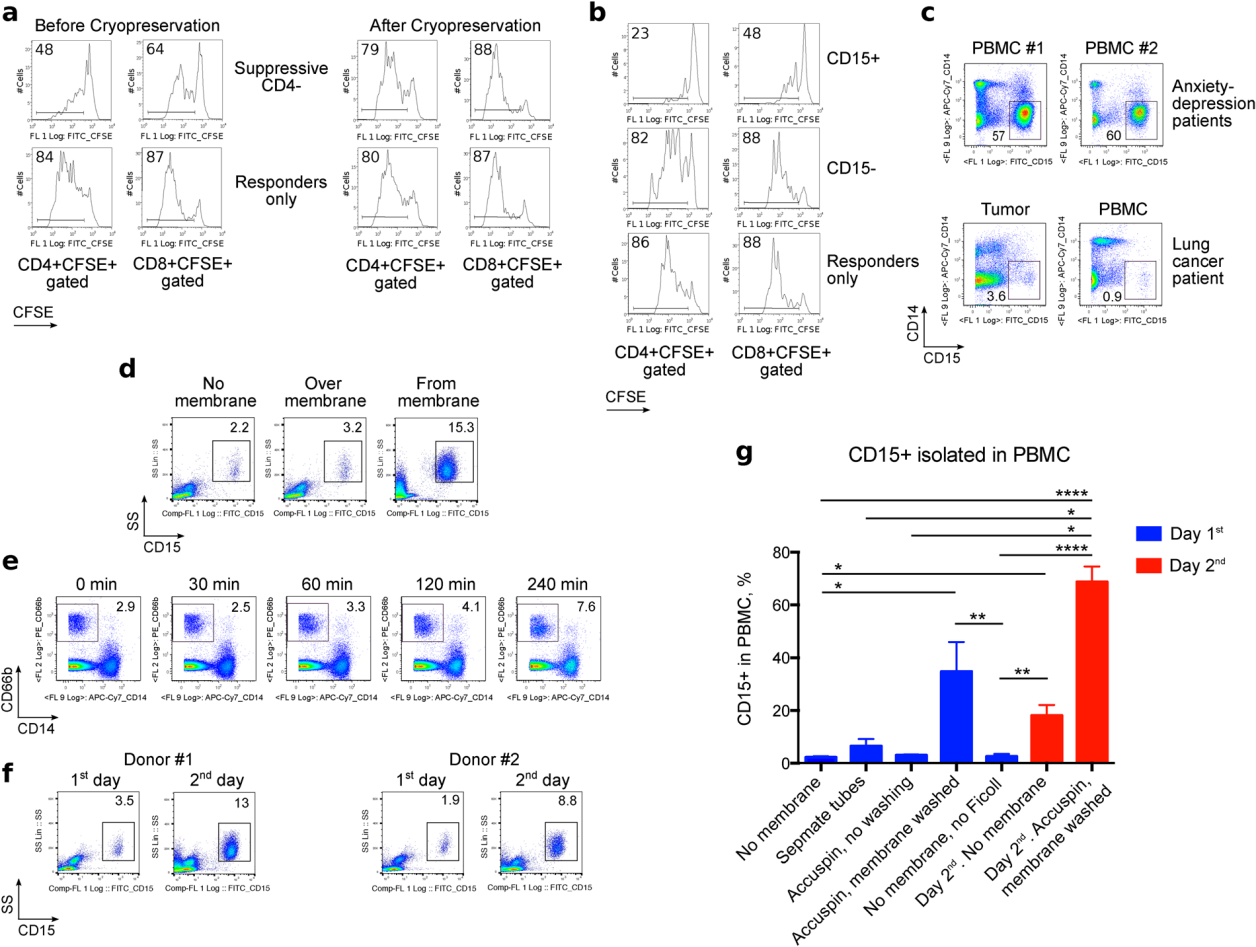
Myeloid origin of CD4^−^ suppressive cells and factors that increase their isolation in PBMC layer. (**a**,**b**) Healthy donor PBMC were stimulated and co-incubated with suppressive cells. (**a**) CD4^−^ were suppressive prior to cryopreservation (left), but not after it (right). Cryopreservation negated suppressive effects of CD4^−^ cells. Data representative of two independent experiments. (**b**) PBMC from a lung transplant recipient were separated into CD15^+^ and CD15^−^ subsets and used as suppressive cells. CD15^+^ cells strongly impaired PBMC proliferation. Data representative of three independent experiments. (**c**) Non-cryopreserved PBMC and tumor single cell suspension samples from a lung cancer patient and 2 anxiety/depression patients showed increased CD15^+^CD14^−^ cells in the anxiety/depression samples. Data representative of four experiments, 11 samples in total. (**d**–**g**) Influence of isolation procedures on myeloid cell populations within PBMC samples. (**d**) Fresh lung transplant blood samples processed within 3 hours after blood draw, over Ficoll (“No membrane”) or in Accuspin tubes, where cells were collected from the PBMC layer exclusively (“Over membrane”) or washed from the membrane (“From membrane”); percent of CD15^+^ in each PBMC sample is indicated. Two experiments with two samples have been performed. (**e**) Whole blood samples were divided into aliquots, incubated on ice for the indicated times, and warmed to room temperature prior to PBMC isolation without membrane tubes. Cooling increased granulocyte contamination within the PBMC layer; 4 samples were evaluated in 3 experiments. (**f**) PBMC were isolated from blood samples within 3 hours of drawing (“1^st^ day”) or on next day (“2^nd^ day”), without membrane tubes. Overnight storage increased the fraction of CD15^+^ cells within isolated PBMC. Data represent 5 experiments with 7 samples. (**g**) Pooled data from 71 blood samples from 47 individuals, including lung cancer (n = 15), lung transplant (n = 22), adult kidney transplant (n = 1), and anxiety/depression (n = 3) patients, as well as healthy donors (n = 6). Data shown as Mean ± SEM (% CD15^+^ in PBMC), with *, **, ***, and **** indicating p < 0.05, p < 0.01, p < 0.001, and p < 0.0001, respectively (Kruskal-Wallis test with Dunn’s Multiple Comparison test). Presented histograms and dot plots consist of 29,263 ± 6,868 (Mean ± SEM) events.

The suppressive effect of CD4^−^ cells was completely abrogated by cryopreservation (Fig. 2a), and this, along with poor CD4^−^ cell survival at the end of the suppression assay (Fig. 1b), suggested a possible polymorphonuclear neutrophil (PMN) origin of the suppressive CD4^−^ cells^12,15,16^. Isolation of CD15^+^ and CD15^−^ fractions from the CD4^−^ PBMC subset and testing them for suppressive activity confirmed the suppressive cells were CD15^+^ PMN (Fig. 2b). As PMN-MDSC were also a promising candidate cell type for the observed effects, and MDSC are commonly associated with malignancies^6–8,17–20^, we evaluated the number of CD15^+^ cells in blood and tumor samples from lung cancer patients. Surprisingly, although we expected MDSC to be isolated on top of the Ficoll gradient, along with lymphocytes, we found that very few CD15^+^ CD14^−^ cells were isolated in lung cancer samples, while blood samples from patients with anxiety/depression were highly enriched with CD15^+^ CD14^−^ cells in PBMC layers (Fig. 2c).

These findings suggested that cell isolation, rather than enrichment of suppressive MDSC, was responsible for the effects observed. In a step-by-step comparison between leukocyte procurement procedures in the 2 different research groups, we identified 3 steps that potentially contributed to an enrichment of CD15^+^ cells. First, use of separation tubes with a membrane (CPT tubes from BD, SepMate tubes from Stemcell Technologies, and Accuspin tubes from Sigma) led to substantial CD15^+^ cell trapping at the surface of gel (CPT), or CD15^+^ cell accumulation on top of plastic (SepMate) or sponge (Accuspin) membranes (Fig. 2d). Second, cooling of blood prior to processing increased the presence of granulocytes in the PBMC layer (Fig. 2e). Third, overnight incubation of whole blood samples increased the CD15^+^ subset recovered with PBMC isolation even if no gel/ membrane tubes were used (Fig. 2f). Overall statistics for the factors affecting CD15^+^ cell isolation in PBMC layers of 71 blood samples are shown in Fig. 2g. Taken together, we found that the suppressive CD4^−^ subset in PBMC consisted predominantly of CD15^+^ PMN cells, which can be enriched in PBMC samples through use of separation tubes, cooling, and/or overnight incubation.

### Suppressive CD15^+^ cells from PBMC isolation have a granulocytic neutrophil phenotype

**Figure 3:**
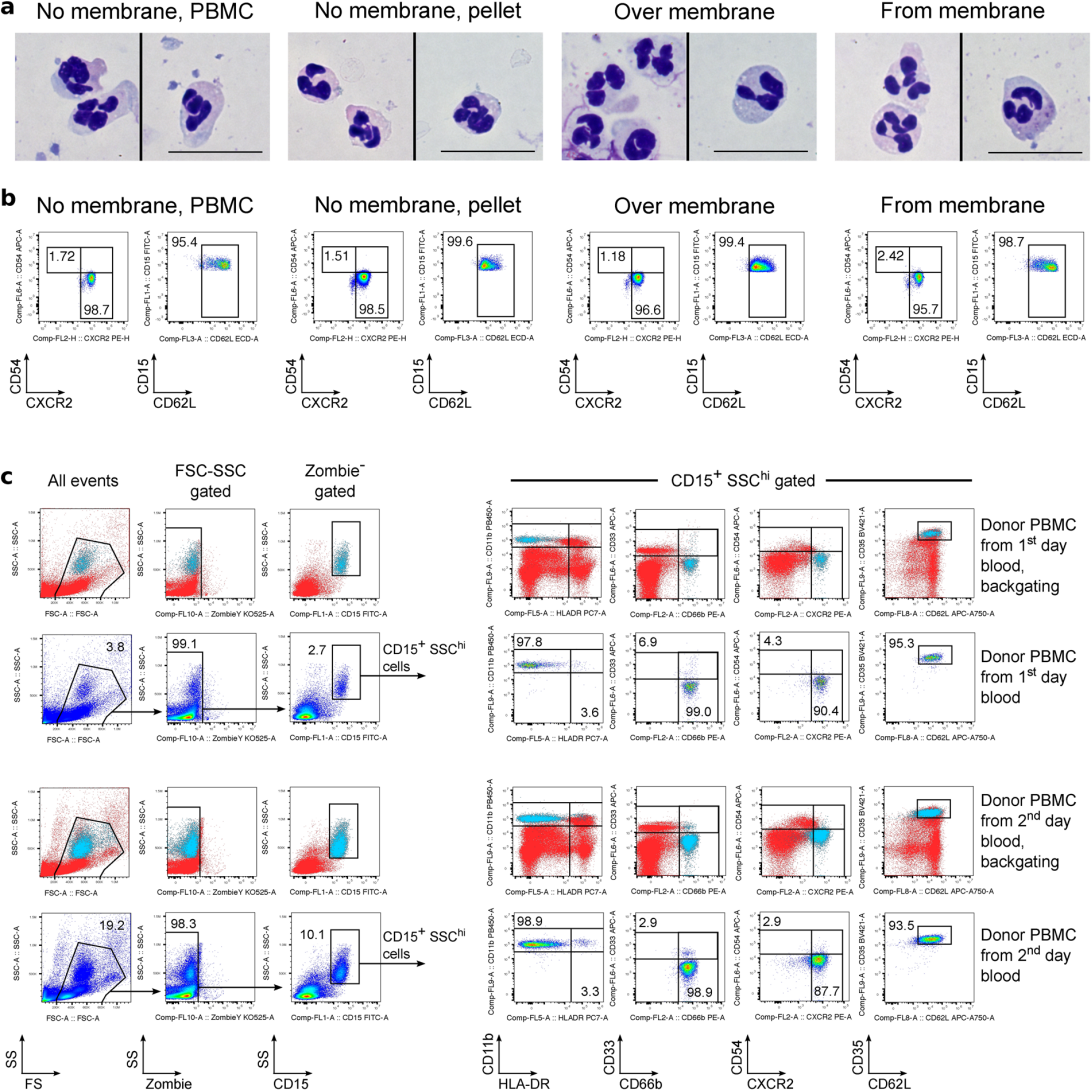
Evaluation of CD15^+^ cell phenotype. (**a**–**b**) PBMC from one day-old healthy donor blood were isolated over Ficoll using conventional tubes (“No membrane, PBMC” and “No membrane, pellet”) or with Accuspin tubes (“Over membrane” and “From membrane”). (**a**) By light microscopy, a typical neutrophilic appearance was seen. (**b**) The same cells as in (**a**) were stained for CD15 and activation markers, CXCR2, CD54 and CD62L. CD15^+^ gated cells are shown, and representative for 7 independent experiments. (**c**) PBMC from healthy donor blood sample were isolated the same day, or next day, over Ficoll using non-membrane tubes. On the left: non-altered, good viability and typical FSC-SSC properties of neutrophils are shown in pseudocolor plots. Additionally, location of CD15^+^SSC^hi^ population of neutrophils is shown by backgating (neutrophils are blue). On the right: 2^nd^ and 4^th^ rows show the full phenotype of gated CD15^+^SSC^hi^ neutrophils, while 1^st^ and 3^rd^ rows identify the same CD15^+^SSC^hi^ population (blue) within the same markers on axes, but compared to the staining of all viable cells (backgating). Presented dot plots consist of 23,728 ± 7,040 (Mean ± SEM) events, not including events in the first column of ungated cells, i.e. left panels of (**c**).

Light microscopy confirmed a granulocytic neutrophil phenotype of the suppressive PMN cells (Fig. 3a). We did not observe signs of neutrophil activation^21,22^ in membrane or non-membrane isolated subsets, or in cells incubated overnight at room temperature or 4 °C, although patients listed for lung transplantion had more heterogeneous expression of activation markers as compared to healthy donors (Fig. 3a–c & Suppl. Figs S2,S3). The full phenotype of suppressive CD15^+^ cells was CD15^+^ SSC^hi^ CD11b^+^ HLA-DR^−^ CD33^−^ CD66b^+^ CD54^−^ CXCR2^+^ CD35^+^ CD62L^+^ CD14^−^ (Figs 2e & 3c). The absence of apparent CD15^+^ cell activation (measured by loss of CD62L or increased CD54), as well as an absence of apoptosis or killing of responder PBMC cells, were consistent with suppression rather than the effects of an oxidative burst, as would be typical of conventional neutrophils^23^. Thus, our CD15^+^ cells appeared like classical MDSC, since they were isolated within the PBMC layer (i.e. they were less dense than normal, pelleted neutrophils). Like MDSC, they demonstrated expression of myeloid and neutrophil markers, and had a typical neutrophil appearance. Furthermore, they exhibited substantial suppression of responder T cells^17,18,20,24–27^. However, the presence of these suppressive cells in most blood samples from transplant recipients and anxiety/depression patients, as well as blood from healthy donors (Suppl. Fig. S4), made this seem an improbable explanation, and led us to further question if an artefact was responsible.

### Suppression by CD15^+^ neutrophils in different stimulatory conditions

**Figure 4:**
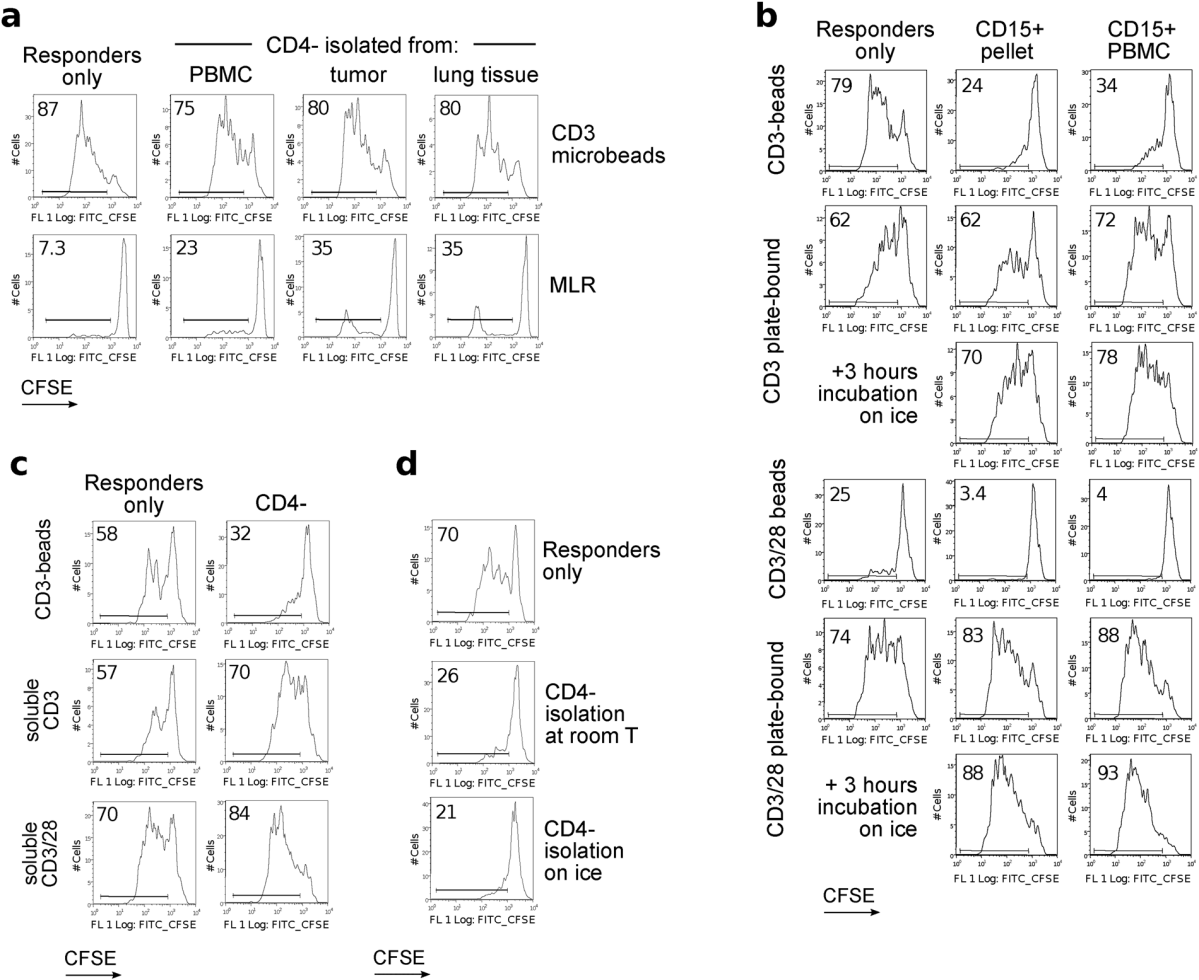
Suppression under different stimulatory conditions. Flow plots showing CFSE-proliferation of CD4^+^ responder cells in suppression assays. (**a**) Comparison of CD3 microbeads stimulation versus MLR with dendritic cells. Data representative of two experiments. (**b**) Comparison of beads vs. plate-bound stimulation. CD15^+^ cells were isolated from PBMC of a patient with anxiety/depression, then used in suppression assay with responder PBMC (1:1 ratio), stimulated either by anti-CD3 microbeads (3.5 beads/cell, top), plate-bound anti-CD3 mAb (0.1 μg/mL, 2^nd^ and 3^rd^ rows), anti-CD3/28 microbeads (0.1 bead/cell, 4^th^ row) or plate-bound anti-CD3/28 (0.1 μg/mL of each, 5^th^ and 6^th^ rows). Additionally, aliquots of CD15^+^ cells were kept on ice for 3 hours prior to use in suppression assays (3^rd^ and 6^th^ rows). (**c**) Comparison of beads vs. soluble antibodies stimulation. CD4^−^ depleted cells from a lung transplant patient were used as suppressors, and responder cells were stimulated either with anti-CD3 microbeads (3.5 beads/cell, top), soluble anti-CD3 mAb (1 μg/mL, middle) or soluble anti-CD3/28 mAb (1 μg/mL, bottom). (**d**) Comparison of suppression by CD15^+^ cells isolated at room temperature vs. on ice. In total, 35 experiments with different types of stimulation and modification of CD15^+^ cells processing were performed, using PBMC from lung transplant (7), anxiety/depression (6), and lung cancer (3) patients, as well as healthy donors (6). Presented histograms and dot plots consist of 1,023 ± 73 (Mean ± SEM) events.

MDSC are known to suppress T cell proliferation induced by anti-CD3 mAb or allogeneic cells. However, our CD4^−^ or purified CD15^+^ cells demonstrated minimal suppression, or even some stimulation, in mixed leukocyte reactions (MLR) (Fig. 4a). Upon further analysis, we found CD15^+^ cells were suppressive only when T cells were stimulated with anti-CD3 or CD3/28 mAb-coated magnetic beads, but not when soluble or plate-bound anti-CD3 or CD3/28 mAb stimulation was used (Fig. 4b,c). Classic CD15^+^ neutrophils, isolated from pellets, also failed to suppress in conditions with soluble or plate-bound antibody stimulation, but did suppress bead-stimulated responder T cells (Fig. 4b & Suppl. Fig. S5). We studied several variables: (i) prolonged incubation of CD15^+^ neutrophils on ice (Fig. 4b,d and Suppl. Fig. S6), (ii) their use when mixed with leftover CD4^−^ cells as we did in previous experiments (Fig. 4c,d & Suppl. Fig. S7), (iii) use of different ratios of CD3 and CD3/28 mAb-coated beads/cell, and (iv) different concentrations of plate-bound CD3 and CD28 mAbs for stimulation (Suppl. Figs S6–S9). Together, our data show that CD15^+^ cells were only suppressive in bead-based stimulation assays.

### CD15^+^ neutrophils interfere with mAb-coated bead cell stimulation

**Figure 5:**
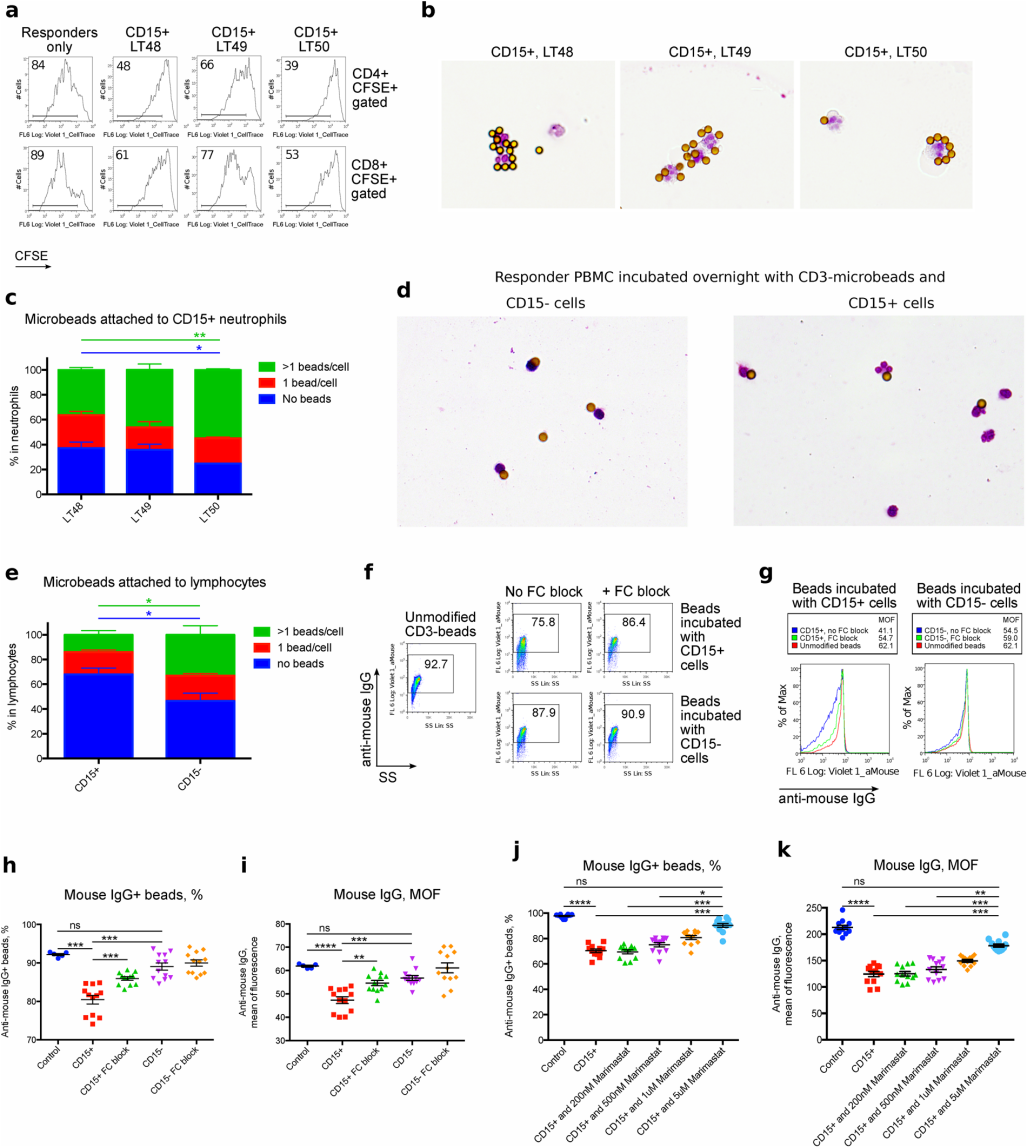
CD15^+^ neutrophils react with magnetic beads. CD15^+^ cells were isolated from PBMC of three lung transplant patients (LT50 was shipped overnight, while LT48 and LT49 were processed within two hours of blood draw). (**a**) Aliquots of the CD15^+^ cells were used for suppression assays, using anti-CD3 microbeads for stimulation (3.5 beads/cell). proliferation of CD4^+^ (top) and CD8^+^ (bottom) responders are shown. (**b**,**c**) CD15^+^ cells were incubated with anti-CD3 microbeads (2 beads/cell) on microscopic slides for 1 hour. (**b**) Cells were then fixed, stained and evaluated by microscopy (×160); demonstrating CD15^+^ cells aggregating with the microbeads. (**c**) Quantification of neutrophil-bead binding (ANOVA). (**d**,**e**) CD15^+^, or CD15^−^ control cells, were incubated overnight with anti-CD3 microbeads and healthy donor PBMC at a 1:1 ratio. (**d**) Microscopic evaluation showed lymphocytes and neutrophils connected with anti-CD3 microbeads (x100). (**e**) Quantification of lymphocyte-bead binding (ANOVA). (**f**–**i**) CD15^+^ and CD15^−^ cells were separated from PBMC and incubated overnight with anti-CD3 microbeads (2 beads/cell) ± Fc-blocking antibodies overnight. Then beads were dissociated from cells, shown by flow cytometry as (**f**) % microbeads positive for anti-mouse IgG or (**g**) mean fluorescence of anti-mouse IgG. Unmodified anti-mouse IgG-stained CD3 beads served as a control. (**h**,**i**) Quantification of (**f**,**g**) pooled from 2 experiments with 4 samples. (**j**,**k**) Rescue of CD3-microbeads from antibody shedding. CD15^+^ cells were incubated overnight with anti-CD3 microbeads (2 beads/cell) in presence or absence of Marimastat. Next day beads were dissociated from cells, and evaluated as (**j**) % microbeads positive for anti-mouse IgG or (**k**) as mean fluorescence of anti-mouse IgG. Unmodified stained anti-CD3 beads served as positive control. Beads co-incubated with CD15^+^ cells without Marimastat served as a negative control. Data combined from 2 experiments with 3 samples. Data shown as mean ± SEM (**h**–**k**) with *, **, ***, and **** indicating p < 0.05, p < 0.01, p < 0.001, and p < 0.001, respectively. (**h**,**j**) Kruskal-Wallis test; (**i**,**k**) ANOVA. Presented histograms consist of 2,146 ± 235 cells (Mean ± SEM), and dot plots analyzing microbeads consist of 32,313 ± 3,712 (Mean ± SEM) events.

As neutrophils are phagocytic, a possible mechanism of suppression might be phagocytosis of the stimulatory microbeads. However, the relatively large bead size (4.5 μm diameter) and our inspection of cell cytospins suggested this was not the case. To further investigate, we isolated CD15^+^ cells from 3 lung transplant patients and divided them into aliquots; some cells were used to confirm their suppressive activity (Fig. 5a), while other cells were incubated on microscope slides to visualize their interactions with anti-CD3 mAb-coated beads, or with anti-CD3 microbeads and healthy donor PBMC responder cells. Within an hour of culture, neutrophils migrated to, and bound the beads, forming rosettes (Fig. 5b). CD15^+^ neutrophils isolated from blood stored for 24 hours collected CD3 mAb-coated beads more rapidly than CD15^+^ cells from freshly processed samples (Fig. 5c), and this enhanced activity correlated with the results of suppression assays (Fig. 5a). On the next day, PBMC responder cells incubated on microscope slides with CD15^+^ cells demonstrated a lack of lymphocytes attached to anti-CD3 mAb-coated beads (Fig. 5d,e). In contrast, more than half of the lymphocytes in PBMC responders, after incubation with control CD15^−^ depleted cells overnight, were attached to at least 1 stimulatory anti-CD3 mAb-coated bead (Fig. 5d,e). These data suggest that CD15^+^ neutrophils interact with CD3 mAb-coated beads and disrupt T cell stimulation.

### CD15^+^ neutrophils cleave antibodies off the surface of mAb-coated beads

We next questioned if the interaction between neutrophils and CD3 mAb-coated beads depended upon Fc recognition. We isolated CD15^+^ cells and, using the CD15-depleted fraction of PBMC as a control, incubated both sets of cells with anti-CD3-coated microbeads, with or without Fc blocking antibodies (we used Fc blocking to prevent recognition of mouse CD3 mAb by neutrophils). Flow cytometry with anti-mouse IgG showed that CD15^+^ neutrophils not only formed rosettes with microbeads, but also cleaved anti-CD3 mAb from the surfaces of microbeads, therefore making beads less active for stimulation of T cells, even when beads were finally released as neutrophils underwent apoptosis 2–3 days later (Fig. 5f–i). ADAM17 is the most abundant human neutrophil sheddase, with various substrate specificities, and Marimastat is a well-known inhibitor of ADAM17 as well as other matrix metalloproteinases^28,29^. We incubated CD15^+^ neutrophils with Marimastat and anti-CD3 mAb-coated microbeads overnight. Marimastat suppressed shedding of antibodies from the surfaces of microbeads in a dose-dependent manner (Fig. 5j–k), confirming that neutrophils use a superficial sheddase to cleave stimulatory antibodies from microbeads.

### Lectins-stimulated NET production by neutrophils is associated with T cells suppression

**Figure 6:**
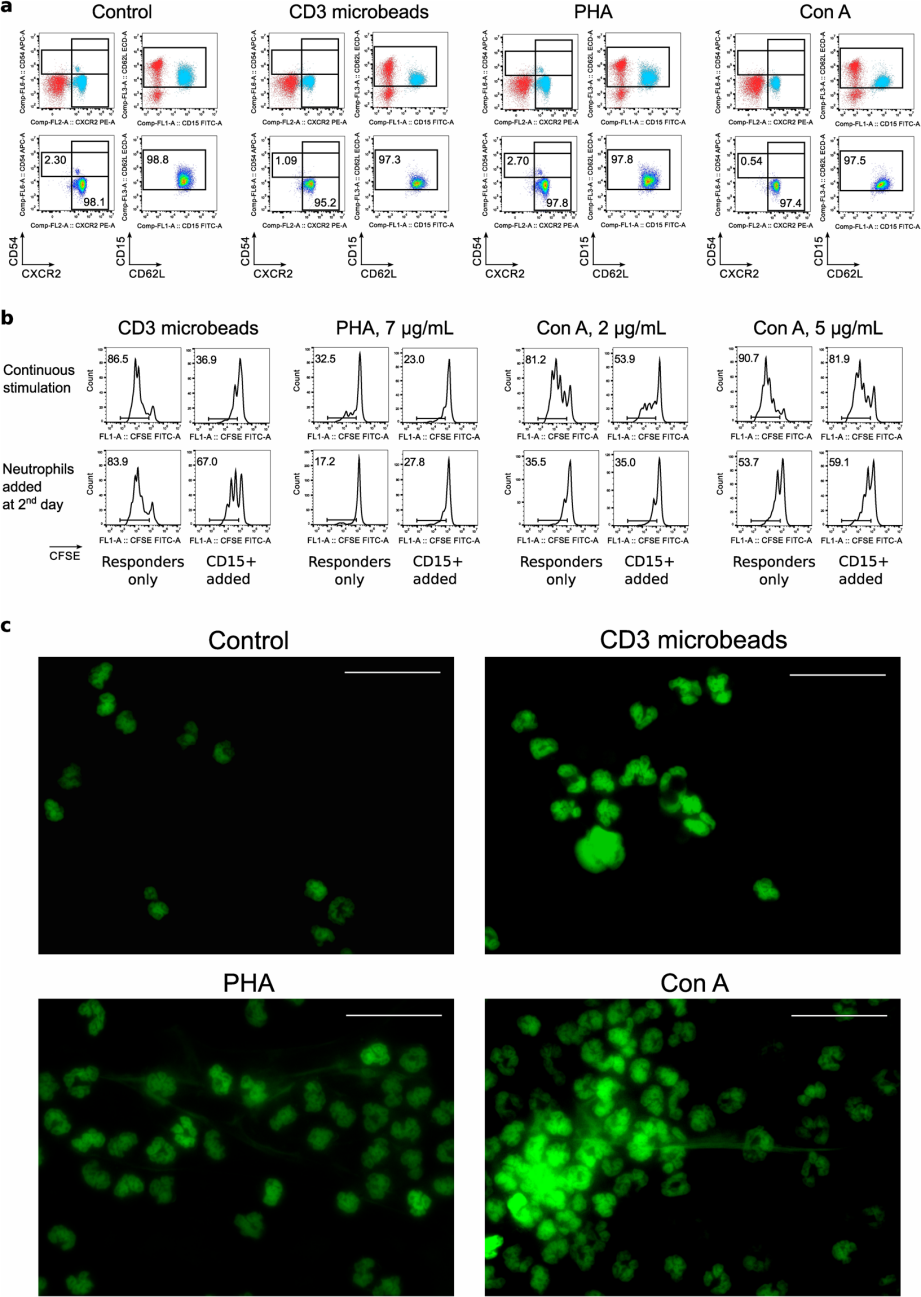
CD15^+^ neutrophils may be directly stimulated by lectins. Healthy donor CD15^+^ cells were incubated for 2 hours at 37 °C in cell culture media in presence of anti-CD3 microbeads (3.5 beads per cell), PHA (7 µg/mL) or Con A (5 µg/mL), then evaluated with flow cytometry for live/dead, CD15 and activation markers: CXCR2, CD54 and CD62L. On top: location of CD15^+^SSC^hi^ population of neutrophils is shown by backgating (neutrophils are blue). On the second row: neutrophils preserve their naïve phenotype. One representative example of cells, isolated the same day as blood drawn, is showed. At least 3 samples from each group, either first day blood or second day blood samples, were evaluated in 5 independent experiments. (**b**) Flow plots showing CFSE-proliferation of CD4^+^ responder cells in suppression assays. Healthy donor CD15^+^ cells were used in suppression assay with CFSE-labeled healthy donor responders PBMC in 1/1 ratios. On top: neutrophils were incubated with PBMC in presence of anti-CD3 microbeads or lectins, and responders showed signs of suppression. On the bottom: same responders PBMC were pre-activated with the same mitogens for overnight, then washed and mixed 1:1 with neutrophils. In absence of lectins, neutrophils had no effect (or even some stimulatory effects) on T cell divisions, but still able to disrupt T cell activation by anti-CD3 microbeads. In total, 3 experiments with 4 healthy donors’ neutrophils and 8 healthy donors’ PBMC responders were performed with similar results. Histograms and dot plots consist of 12,695 ± 2891 (Mean ± SEM) events. (**c**) Microscopic evaluation of CD15^+^ cells after 1 hour incubation with anti-CD3 microbeads, PHA or Con A. Sytox green stained cells showed an absence of aggregation and NET production in control condition, but signs of aggregation and NET production with both lectins. Anti-CD3 microbeads, as it was shown in light microscopy, were aggregated with neutrophils and may be seen as round unstained shapes. One experiment out of two is shown. More details are shown in Suppl. Figures 10–15.

To study the effects of neutrophils on the suppression assay when responder cells were stimulated with non-specific activators of T cells, we tested two classical mitogenic lectins, PHA and Con A. At the tested concentrations of both mitogens, as well as CD3 microbeads, healthy donor CD15^+^ cells remained naïve and viable (Fig. 6a & Suppl. Fig. S10). However, to our surprise, assays with neutrophils and responder cells, stimulated with either lectin, showed evident suppression (Fig. 6b, top row and Suppl. Figs S10,S11). We considered that mitogenic lectins may directly affect neutrophils, as described^30–33^, but there were no alterations of neutrophil phenotype (CD62L, CXCR2 and CD54) in our experimental conditions.

To dissect direct stimulatory effects of lectins on T cells *versus* neutrophils, we pre-stimulated responder PBMC overnight with lectins, washed, and added neutrophils the next day. This approach completely abrogated any suppressive effects in assays with lectins but not anti-CD3 microbead stimulation (Fig. 6b, bottom row and Suppl. Figs S10,S11). Finally, since neutrophils can undergo aggregation due to capping by lectins^30–33^, and rapid neutrophil extracellular trap (NET) production without neutrophil death or ROS production is reported^34^, we performed microscopic evaluation of lectin-activated neutrophils. Lectin-stimulated neutrophils, but not control cells or neutrophils co-incubated with CD3-microbeads, rapidly aggregated, and formed nucleic acid-stained strings, presumably NET structures (Fig. 6c and Suppl. Figs S12–S15). As NETs contain proteolytic enzymes^34^, the activity of those enzymes may be detrimental for T cell stimulation, and NET production may be a plausible mechanism for the suppressive effects of lectins.

In conclusion, we describe a number of potential sources of artefact in studies of human MDSCs. First, there is substantial contamination of PBMC samples with conventional CD15^+^ neutrophils. This contamination occurred in aged blood, in cooled blood, and in blood processed with membrane/gel tubes, but not in cryopreserved PBMC. Second, human conventional neutrophils can suppress T cell proliferation induced by anti-CD3 or anti-CD3/28 mAb-coated microbeads, due to cleavage of the stimulating antibodies from the activating beads, and they can suppress T cell proliferation induced by lectins due to induction of NETs on the CD15 + cells. This artefactual “suppression” closely mimics the characteristics of PMN-MDSC. Therefore, the results of studies reporting suppressive activity of human PMN-MDSC, when performed with microbead or lectin stimulation, need to be interpreted with caution.

## Discussion

There is widespread interest in characterization of immune cells with suppressive actions, given their potential use for cell therapy in transplantation and autoimmunity, or conversely, as therapeutic targets in cancer patients. These applications depend upon careful analysis of each suppressive cell population. Here, we demonstrate some important and surprising features of normal human neutrophils, including that they may be isolated within the PBMC layer, and that they can suppress T cell proliferation induced using mAb-coated bead or lectin stimulation.

It is known that low density neutrophils can be isolated in the PBMC layer of samples collected from patients with certain pathological conditions^10,18,25,26,35^. However, our data show that membrane or gel containing tubes lead to trapping of naïve, normal (not low) density neutrophils above the membrane, along with PBMC. The extent of neutrophil contamination of PBMC samples depends on the conditions of blood processing and can reach as much as 70% of the total PBMC fraction. Recognition of such contamination with normal density neutrophils is important to avoiding erroneous conclusions about the nature of suppressive cells.

Our studies suggest that PBMC isolated in tubes with gels or membranes, compared with PBMC isolated over Ficoll with conventional tubes, may provide artificially increased neutrophil-related proteins, enzymes, RNAs, etc. Similar artefacts will arise in comparisons of freshly isolated versus cryopreserved PBMC, since neutrophils typically do not survive freezing and thawing. Although researchers are usually aware of cryopreservation-related differences, and therefore do not use fresh and cryopreserved cells for direct comparison in functional studies, these cells may be used for protein isolation, for nucleic acids purification and other techniques, and neutrophil-rich non-cryopreserved samples may be a source of significant artefact. Another set of problems can arise from using magnetic bead-based cell isolation with blood cells. Thus, commercial kits use a depletion strategy to isolate ‘untouched’ cells of interest, wherein unwanted PBMC components are labeled with corresponding antibodies in pre-mixed depleted cocktails, but do not allow for 50–70% of neutrophils in the starting PBMC population, and the purity of the resulting population may be significantly affected (our unpublished observations). To solve this problem, investigators may need to add anti-CD15 beads or anti-CD15 antibodies into their depletion step. It is also important to note that it is challenging to diagnose neutrophil contamination retrospectively, because cryopreserved PBMC samples will show an absence of CD15^+^ cells by flow cytometry.

We have observed suppressive effects by conventional CD15^+^ neutrophils in each of our test groups, including healthy donors, patients with anxiety/depression, patients listed for lung transplantation, patients who have received kidney, liver or lung allografts, and patients with lung cancer, as well as in adult and pediatric populations. Thus, the findings we describe are not specific to immunosuppressed, aged or tumor-bearing populations, but rather, appear universal. We caution that the suppressive artefact may be relevant to virtually any studies of human blood cells that involve stimulation and/or suppression *in vitro*, if microbeads or lectins are used, and if researchers do not monitor their cells for CD15^+^ neutrophil contamination. Thus, expansion of T cells, manufacture of chimeric antigen receptor T cells, Treg suppression assays with CD3-depleted PBMC as antigen-presenting cells, cytokine production tests, Th1/Th2/Th17/iTreg conversion studies, and rapid stimulation to evaluate signaling events are just a few examples of the studies which may be significantly affected by neutrophil contamination.

Prior studies indicated that human neutrophils in specific conditions, or with specific phenotypes, could suppress T cell proliferation^19,26,35–39^, but to our knowledge, “suppression” by normal, unactivated CD15^+^ neutrophils is unreported. Of note, our initial studies using activating beads showed suppression that closely mimicked the properties of neutrophil MDSC; and only additional studies with different stimulatory conditions (MLR, plate-bound vs. soluble antibody stimulation) allowed us to identify the role of CD15^+^ neutrophils. We are unaware of any published studies that used plate-bound antibodies or IgG (except for anti-CD18^40^ and anti-TREM1^41^) to stimulate neutrophils, while plate-bound immune complexes have served as a tool in neutrophil studies for decades. Our data indicate that plate-bound antibodies, other than when present in immune complexes, do not efficiently activate neutrophils via Fc-receptor stimulation.

Why CD15^+^ neutrophils suppress T cell responses to mAb-coated beads but not corresponding mAb-coated plates is unknown. We hypothesize that plate-bound CD3 and/or CD3/28 antibodies cover a relatively large area on plastic, such that the levels of neutrophil produced sheddases may be far too dilute to have an effect, whereas microbeads form rosettes with neutrophils, as we have shown, allowing for much higher local concentrations of proteolytic enzymes. Neutrophils may also physically restrict the direct contact of T cells with microbeads, but not with plate-bound T cell stimulation. By contrast, the unexpected findings of NET production by lectin-stimulated neutrophils provide yet another means by which these cells can artefactually impair T cell proliferation.

In a broader context, our data raise concerns to the validity of some human MDSC studies, in addition to queries raised by others^9,10,27^. As shown here, human conventional neutrophils can be isolated within the PBMC layer, have a naïve phenotype, and yet suppress T cell proliferation (through artefactual mechanisms). Thus, these 3 factors cannot be used as sufficient determination for human PMN-MDSC. We suggest that all studies evaluating human MDSC should confirm suppressive properties of their myeloid cells using functional tests like MLR or by assays where plate-bound or soluble antibody stimulation is used, and conclusions should not be based solely on mAb-coated microbead-based or lectin-based stimulation assays.

## Methods

### Donors

Blood samples were obtained from healthy volunteer donors (n = 11) via the UPenn Human Immunology Core (PBMC), or from controls during a generalized anxiety disorder study as controls (whole blood, n = 7). All donors signed an informed consent.

### Patients

We evaluated blood from pediatric liver (n = 21) or kidney (n = 8) transplant (Tx) recipients, as described^42^, as well as from adult liver (n = 7) and kidney (n = 7) transplant recipients. Patients received standard triple therapy of calcineurin inhibitor, corticosteroids and an antimetabolite. In adults, we collected blood pre-transplant, in the first week post-transplant, at 3 months and 1 year post-transplant, whereas in children, blood was collected only from patients who had stable graft function and no episodes of rejection during the prior 6 months. Enrolled lung transplant patients (n = 24) were described previously^43^. Lung cancer patients (n = 16) underwent surgical resection and provided portions of tumor tissues and blood samples, as described^44^. We also assessed blood samples from 9 patients with generalized anxiety disorder (anxiety/depression)^45^. In each case, specimens were collected according to a protocol approved by the corresponding Institutional Review Board.

### Lymphocyte isolation from tumors

For tumor samples, we used methods that were recently described^44^. In brief, tumors were trimmed, sliced into small pieces, and digested for 45–95 min with shaking. The enzymatic cocktail for tumor digestion consisted of serum-free Hyclone Leibovitz L-15 medium supplemented with 1% penicillin-streptomycin, collagenase type I and IV (170 mg/L = 45–60 U/mL), collagenase type II (56 mg/L = 15–20 U/mL), DNase I (25 mg/L) and elastase (25 mg/L), all from Worthington Biochemical.

### PBMC and neutrophil isolation

For liver and kidney allograft recipients, we used PBMC isolated the same day with CPT tubes (Cell Preparation Tubes, BD Bioscience) and shipped overnight, as described^42^. When indicated, blood samples were shipped overnight at room temperature, and PBMC were isolated using tubes from SepMate (StemCell Technologies) or Accuspin (Sigma-Aldrich), using centrifugation (400 g, 35 minutes) over Ficoll-Paque PLUS (GE Healthcare) at room temperature, as higher speeds listed in the corresponding manufacturer’s protocol led to significant hemolysis. When indicated, we used a standard Ficoll-Paque PLUS protocol. In some experiments, we collected pellets containing RBC and neutrophils, and enriched with neutrophils using RBC Lysis Buffer (Santa Cruz Biotechnology).

### Functional studies

T-regulatory (Treg) cell isolation and suppression assays were performed as described^14,42^. We used CD4**-**depleted PBMC or CD15^+^ isolated cells (CD15^+^ microbeads, Miltenyi Biotec) instead of Treg cells, with Tregs as a positive control for suppression in the same experiments. Aliquots of PBMC from two healthy donors (Table 1) were used as standardized responder cells. Cells were stimulated with CD3 mAb-coated microbeads (OKT3 mAb-coated M-450 Dynabeads) unless specified (Suppl. Table S1). MLR stimulation assay with irradiated, mature, monocyte-derived DCs from unrelated healthy donors (as described^44^) were mixed with PBMC responders to provide stimulatory and co-stimulatory signals. In suppression assays involving stimulation with lectins, Con A and PHA, we studied aliquotes of PBMC of 9 unrelated healthy donors.

For suppression assays, CD4^−^ or CD15^+^ isolated suppressor cells were mixed at 1:1 ratios with CFSE-labeled healthy donor PBMC (responders) and incubated for 4 days. Cell culture media consisted with RPMI 1640 (Invitrogen), supplemented with 10% heat-inactivated FBS, penicillin and streptomycin, and 2-mercaptoethanol. Viability of CD15^+^ cells evaluated in all suppression assays was >90%, according to Trypan Blue or PI staining. In some experiments, when indicated, PBMC were activated with CD3 microbeads or lectins for overnight and washed the next day, then neutrophils were added for 3 days.

To assess cell interactions with microbeads, isolated CD15^+^ cells or CD15^−^ controls were incubated with beads on sterile microscope slides in cell culture media for specified times, then fixed and stained with Kwik-Diff kit (Thermo Fisher Scientific). Neutrophils and lymphocytes were identified by their typical morphology. Numbers of neutrophils or lymphocytes with 0, 1 or >1 attached microbeads were assessed independently by 2 researchers, using multiple microscopic fields. Counting ended when the total cell number reached 200 cells of any type or when the whole slide was evaluated.

To study microbeads, CD15^+^ cells or CD15^−^ controls were incubated overnight with anti-CD3 beads, with or without Fc block, and non-toxic concentrations (5 μM and below) of Marimastat, an inhibitor of ADAM17 and other matrix metalloproteinases (Santa Cruz Biotechnology), were used. On the next day, beads were completely dissociated from cells by vortexing and pipetting (Suppl. Fig. S16), and beads were stained with Pacific Blue Goat Anti-Mouse IgG (Molecular Probes, cat #P31582) to evaluate residual attachment of anti-CD3 mAb by flow cytometry.

To evaluate NET production, CD15^+^ cells were isolated with magnetic beads, and incubated for 1 hour at 37 °C in cell culture media on sterile microscopic slides in CO2 incubator in presence of CD3 microbeads (3.5 beads per cell), PHA (7 µg/mL) or Con A (5 µg/mL). Then slides were washed, fixed in 4% formaldehyde and stained with Sytox green (1μM) or DAPI (Mounting Medium with DAPI, 4′,6-diamidino-2-phenylindole).

### Flow cytometry

Cells were stained for live/dead fixable reagent, then washed, and FC blocking reagent (Human TruStain FcX, Biolegend) was applied for 5–10 min at room temperature. Then we labeled cells with corresponding superficial antibodies (Supplemental Table S1) for 30 minutes in pre-titrated concentrations. To evaluate Caspase-3, cells were permeabilized and fixed, then stained for active Caspase-3 according to manufacturer protocol (BD bioscience, catalog #550914). We evaluated cells using CyAn Dako or CytoFLEX flow cytometers and analyzed data with FlowJo. Compensation was performed using single stains and fluorescent minus one (FMO) controls. We applied gating on cells negative for live/dead fixable reagent to exclude dead, apoptotic and non-hematopoietic cells and therefore to markedly decrease non-specific signals. The examples on full gating strategies are shown at Fig. 3c. Viability of CD15^+^ cells evaluated in all phenotypic flow cytometry experiments was >90% even after overnight incubation, as illustrated at Fig. 3c. In our experiments performed without CD15^+^ isolation, conventional neutrophils were not prone to apoptosis for the first two days.

### Statistical analysis

We used GraphPad Prism 6.0 software. All data were tested for normal distribution of variables. Measurements between two groups used Student-t test if normally distributed, or Mann-Whitney U test if otherwise. Groups of 3 or more were analyzed by 1-way analysis of variance (ANOVA) if normally distributed (with Tukey’s multiple comparison test), or if not, by Kruskal-Wallis test with Dunn’s multiple comparison tests. We used Pearson correlation for normally distributed data. For 2 × 2 contingency tables, we used Fisher’s exact test. In experiments with cells cultured on microscopic slides, we counted number of cells in each category, then calculated the fractions of total with 95% confidence intervals, and then applied those means with 95% CIs as means ± SEM in 2-way ANOVA analysis as indicated.

### Data availability

The datasets generated during and/or analyzed during the current study are available from the corresponding author on reasonable request.

## Electronic supplementary material

Supplementary information
