# Supplementary material for: Human neutrophils can mimic myeloid-derived suppressor cells (PMN-MDSC) and suppress microbead or lectin-induced T cell proliferation through artefactual mechanisms
Source: Sci Rep. 2018 Feb 16;8:3135. doi: 10.1038/s41598-018-21450-6 (PMC5816646; doi:10.1038/s41598-018-21450-6)
Supplement: Supplementary file 1 — Supplementary information [file 41598_2018_21450_MOESM1_ESM.pdf]

SUPPLEMENTAL INFORMATION

Human neutrophils can mimic myeloid-derived suppressor cells (PMN-MDSC) and suppress microbead or lectin-induced T cell proliferation through artifactual mechanisms

Negorev D. at al.

Dmitri Negorev, Ulf H. Beier, Tianyi Zhang, Jon G. Quatromoni, Pratik Bhojnagarwala, Steven M Albelda, Sunil Singhal, Evgeniy Eruslanov, Falk W. Lohoff, Matthew H. Levine, Joshua M. Diamond, Jason D. Christie, Wayne W. Hancock and Tatiana Akimova*

Table S1. Antibodies and reagents used for flow cytometry and cell stimulation

**Flow cytometry**				
**#**	**Name**	**Clone/catalog #**	**Color or Type, Ex/Em**	**Manufacturer**
1	CFSE	cat# C1157	492/517 nm	ThermoFisher Scientific
2	CellTrace	cat#C34571	405⁄450, Violet	ThermoFisher Scientific
3	Live/dead	cat# L34957	Aqua, 405/525 nm	ThermoFisher Scientific
4	Zombie	cat# 423103<br>cat#423101	Yellow 405/572,<br>Aqua 405/516	Biolegend
5	CD4	SK3	APC, Pacific Blue, APC-Cy7	Biolegend
6	CD11b	M1/70	Pe-Cy7	Biolegend
7	CD14	HCD14	APC-Cy7, PerCP	Biolegend
8	CD15	HI98	FITC, PE	Biolegend
9	CD33	P67.6	APC	Biolegend
10	CD35	E11	BV421	BD Biosciences
11	CD54	HCD54	APC	Biolegend
12	CD66b	G10F5	PE	Biolegend
13	CD62L	DREG-56	Pe-Cy5, APC-Cy7, PE-Dazzle594	BD Biosciences, Biolegend
14	CD95 (Fas)	DX2	Pe-Cy7	Biolegend
15	Caspase-3, Active Form	C92-605	PE	BD Biosciences
16	CXCR2	5E8/CXCR2	PE	Biolegend
17	HLA-DR	307616	Pe-Cy7	Biolegend
**Stimulation**				
18	CD3 mAbs-coated beads	OKT3 and Dynabeads	MACS GMP pure and<br>M-450 Tosylactivated beads	Miltenyi Biotec and ThermoFisher Scientific
19	CD3/28 beads	11131D	Human T-Activator CD3/CD28	ThermoFisher Scientific
20	CD3	OKT3	LEAF™ Purified	Biolegend
21	CD28	CD28.2	LEAF™ Purified	Biolegend
22	Concanavalin A	C5275-5MG	Aseptically processed	Sigma-Aldrich
23	Phytohemagglutinin	0215188401	Powder	MP Biomedicals

Supplemental Figures

Figure S1: Different requirements of CD3 microbeads stimulation between PBMC of two healthy donors

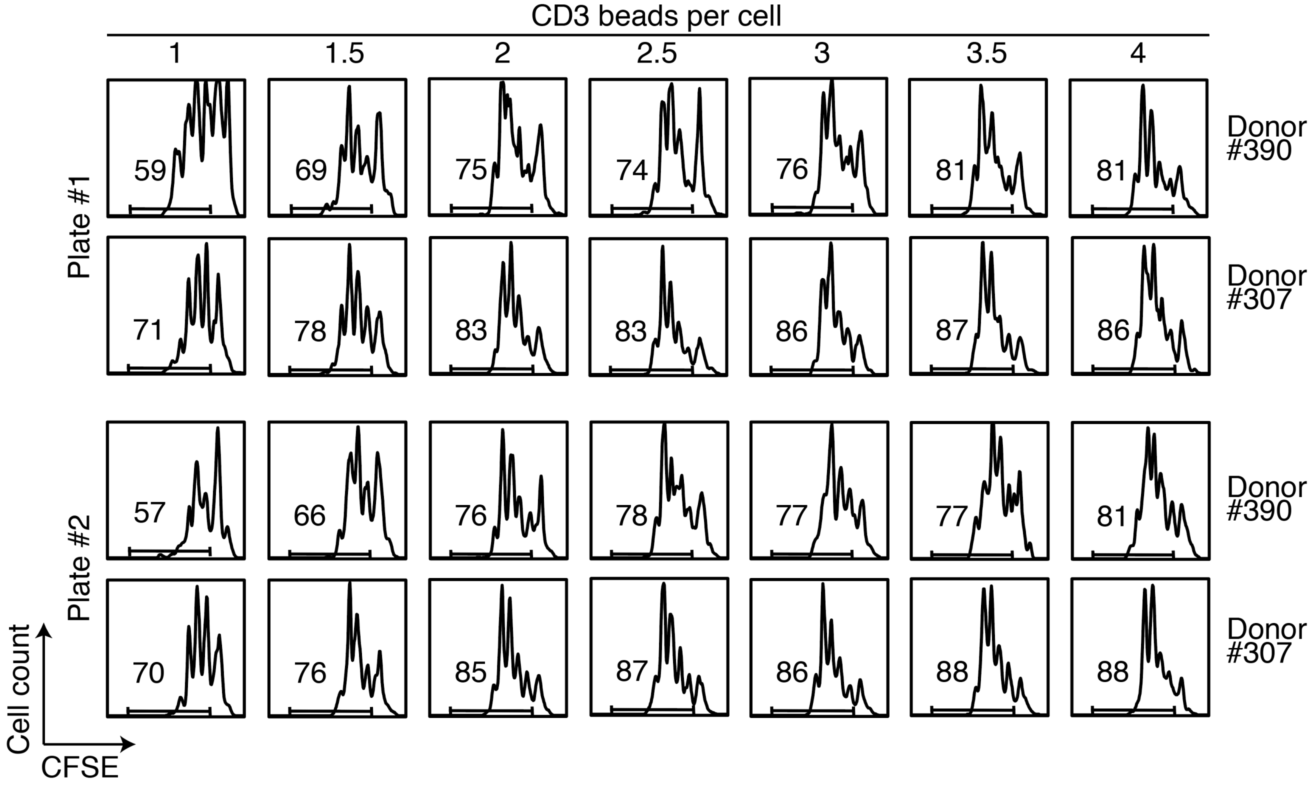

PBMC cells from donors #390 and #307 were mixed with corresponding numbers of CD3 microbeads (indicated) and incubated for 4 days as in suppression assay. Divisions of $CFSE^{+}CD4^{+}$ cells from PBMC #307 peaked at a concentration of 2 beads per cell, whereas PBMC #390 required stimulation with 3.5–4 CD3 microbeads per cell to reach maximal division. One experiment out of three is shown. Presented histograms consist of 661 ± 73 (Mean ± SEM) events.

Figure S2: Phenotype of CD15$^+$ cells, isolated by different methods, immediately and the next day

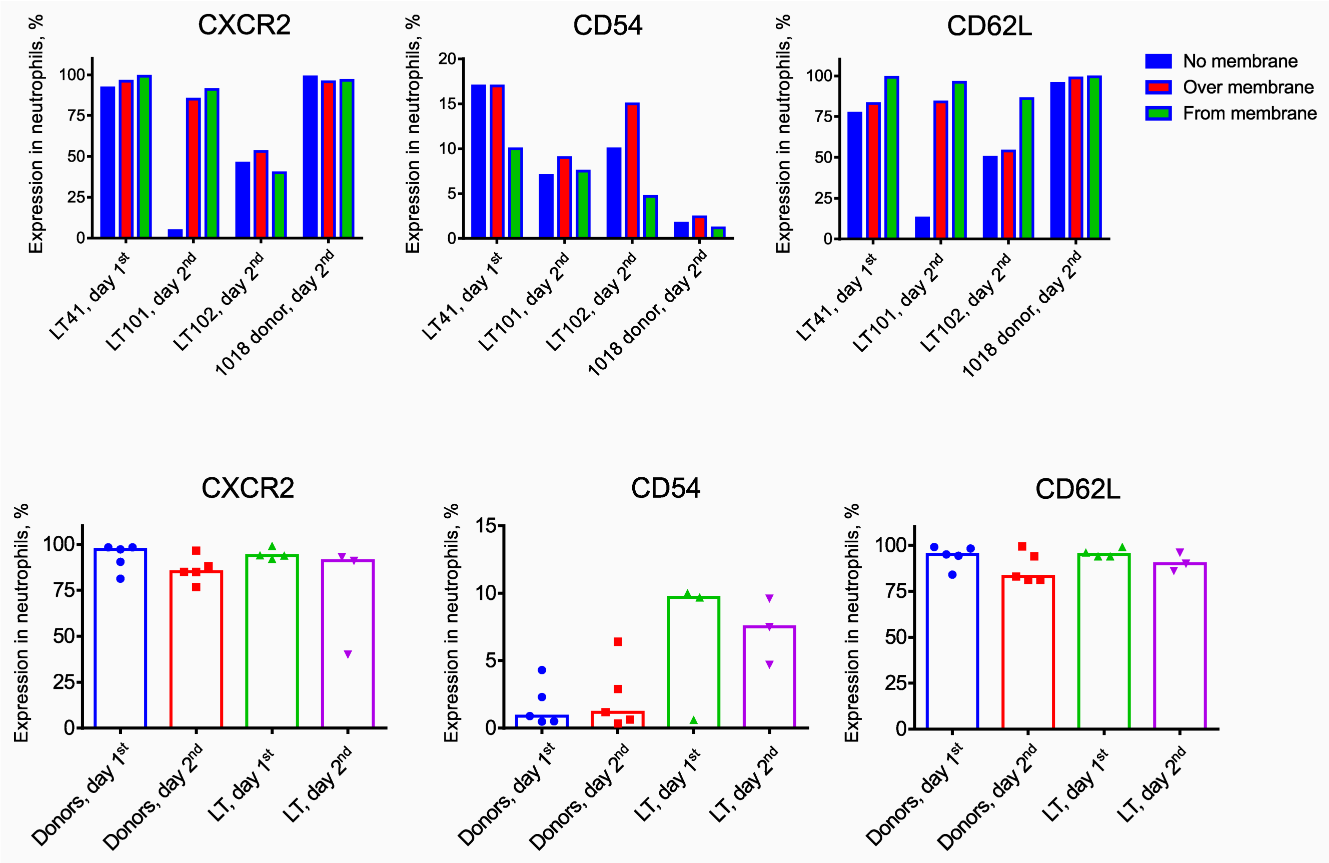

(A) PBMC were isolated from the blood samples over Ficoll using conventional tubes ("No membrane") or with membrane-containing tubes ("Over membrane" and "From membrane"). Then cells were stained for live/dead, CD15 and activation markers, CXCR2, CD54 and CD62L. Bars illustrate an absence of activation of neutrophils, isolated with membrane-containing tubes, in comparison with conventional Ficoll isolation. (B) PBMC were isolated from healthy donors ("Donors") or from patients listed for lung transplant ("LT"), immediately or the next day. In comparison with donors, LT neutrophils tend to express more CD54. At the second day blood, neutrophils tend to moderately decrease CD62L and CXCR2 expression, but no apparent signs of activation were observed. At least 3 samples in each group were evaluated in 14 independent experiments. Medians and individual values are shown.

Figure S3: No substantial changes in phenotype of CD15$^+$ cells after overnight incubation of the whole blood at 4˚C

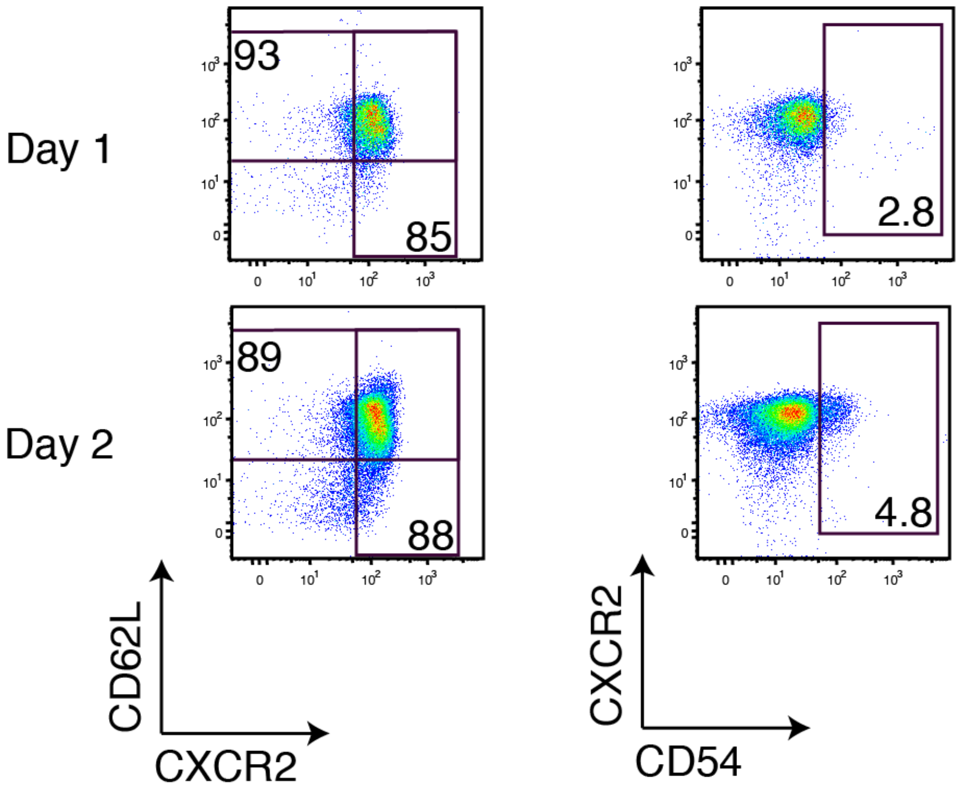

PBMC were isolated from the same blood sample within 3 hours of drawing (top) or on next day (bottom) after incubation at 4˚C, and an absence of activation of neutrophils was confirmed by flow cytometry: CD15$^+$ cells continued to express CD62L and CXCL2 and did not upregulate CD54. One experiment out of three is shown. Presented dot-plots consist of 11,321 events (top) and 32,185 events (bottom).

Figure S4: CD15$^+$ cells from healthy donors are suppressive

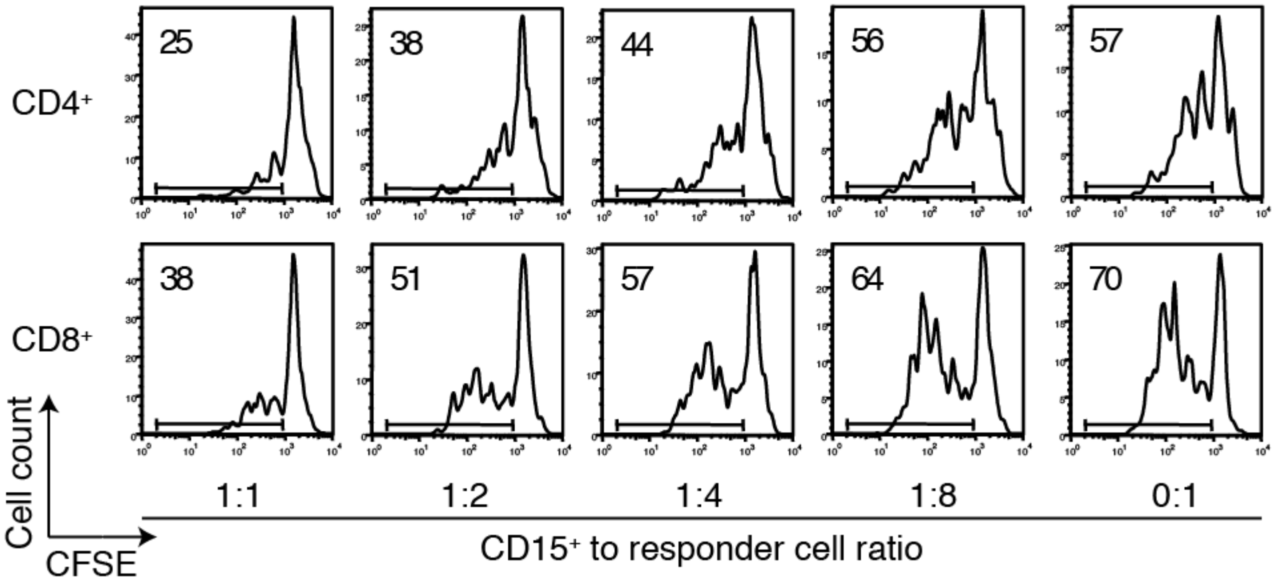

PBMC were isolated with SepMate tubes, CD15+ cells were isolated with magnetic beads, and incubated with healthy donor (#390) responder cells in suppression assay. One representative experiment out of six is shown, and all tested healthy donor CD15$^+$ cells were suppressive. Four of them had their PBMC isolated the same day with SepMate or Accuspin tubes, and in 2 cases PBMC were isolated one the day after acquisition. Presented histograms consist of 1,201 ± 46 (Mean ± SEM) events.

Figure S5: CD15$^+$ are only suppressive in microbead-based stimulation assays

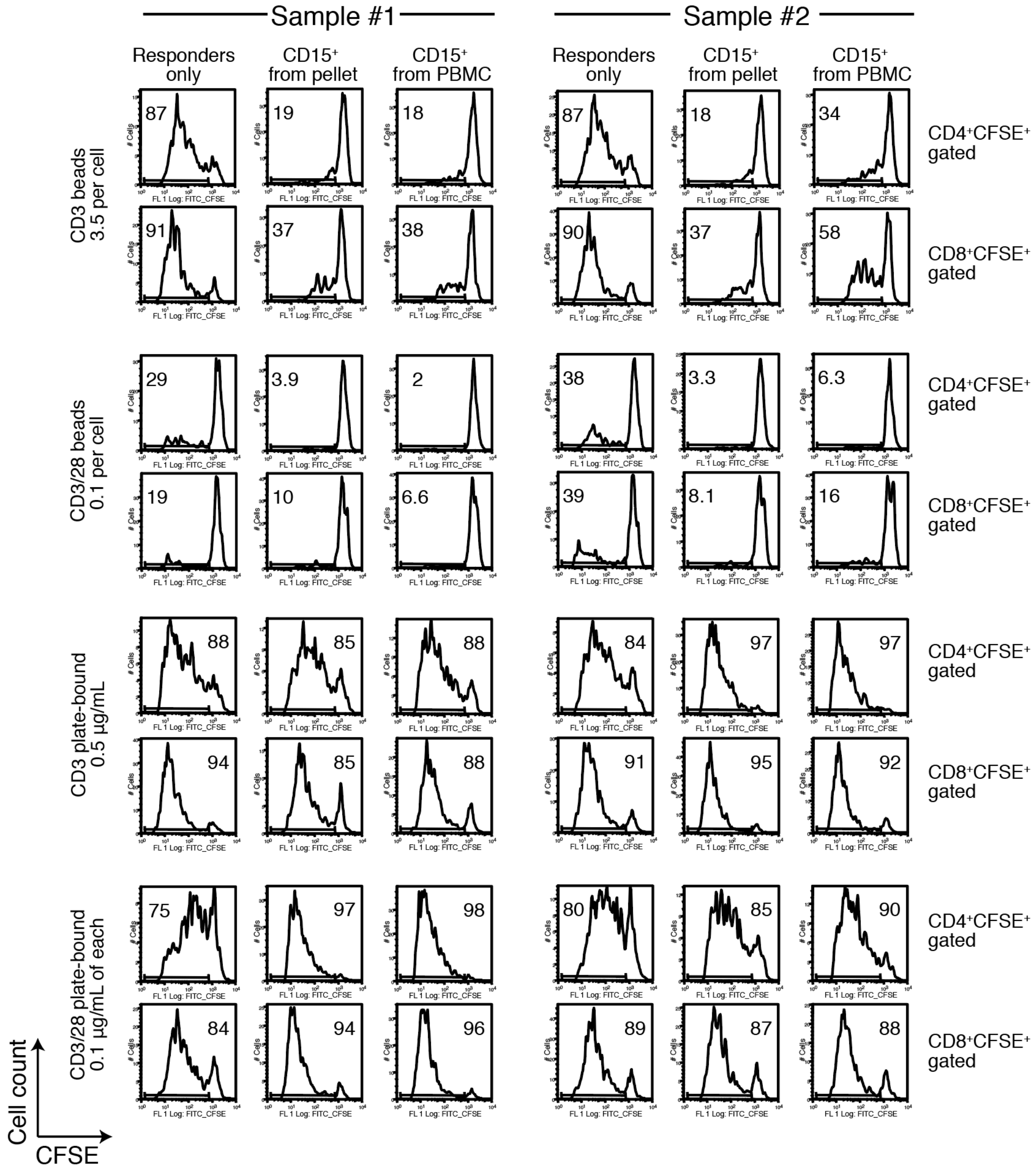

CD15$^+$ cells were isolated with magnetic beads from PBMC and from cell pellets (under Ficoll). CD15$^+$ cells from 1 lung transplant patient (sample #1, on the left) and one healthy donor (sample #2, on the

right) were incubated with healthy donor ID #390 responder cells in suppression assay. Responder cells were stimulated with CD3 microbeads, CD3/28 microbeads, with CD3 plate-bound mAbs or CD3 & CD28 plate-bound mAbs, as indicated. $CD15^+$ cells were suppressive only in experiments with microbead stimulation. Results show 1 of 8 experiments with CD3 or CD3/28 microbeads vs. plate-bound antibody stimulation, 1 out 4 experiments with CD3 versus CD3/28 microbeads, and 1 of 2 with PBMC $CD15^+$ vs. pellet $CD15^+$ cells. In total, 10 patients (6 with anxiety/depression, 3 lung transplant and 1 lung cancer) and 5 healthy donor samples were evaluated. Different concentrations of plate-bound antibodies and bead-to-cell ratios were tested, and suppression was only seen in microbead-stimulated assays. Presented histograms consist of 1,256 ± 40 (Mean ± SEM) events.

Figure S6: Prolonged incubation of PBMC on ice and use of different concentrations of plate-bound CD3 mAb in suppression assay

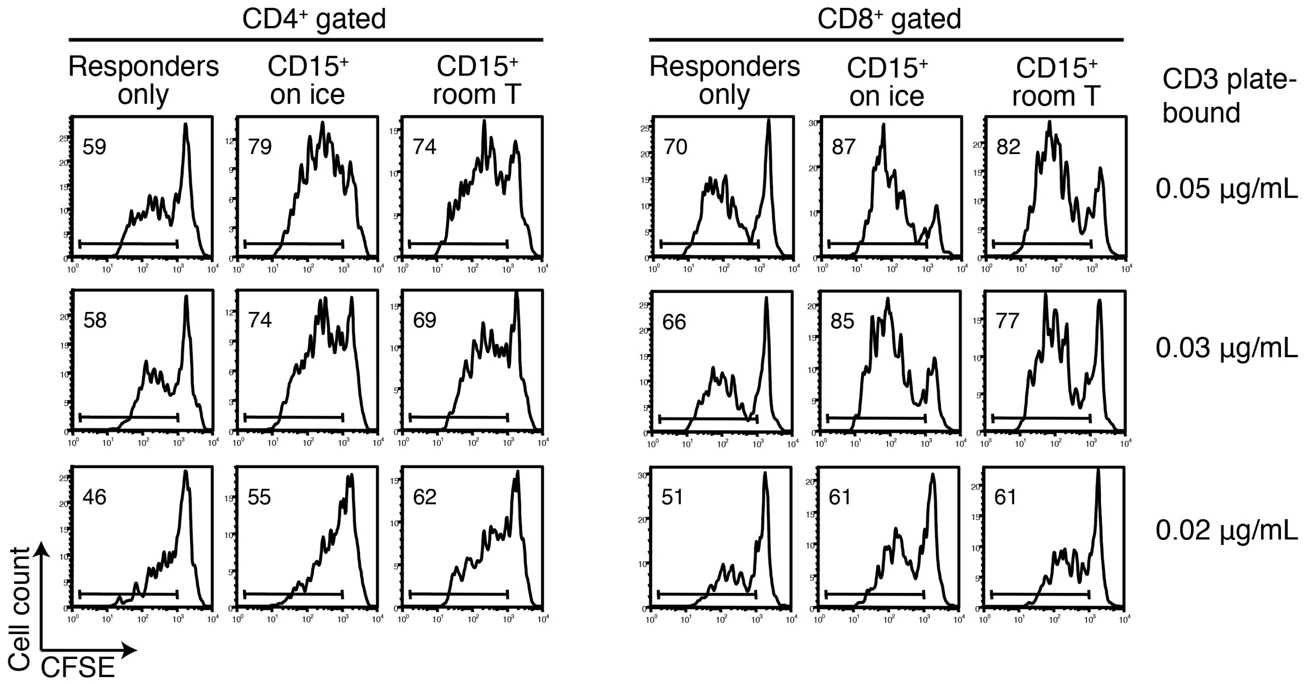

CD15^{+} cells were isolated with magnetic beads, from 1st day PBMC of lung transplant patient, at room temperature or on ice, and incubated with healthy donor (#390) responder cells in suppression assay. Responders were stimulated with indicated concentrations of plate-bound CD3 mAbs or by CD3 microbeads (as shown at Figure 4D). One experiment from two is shown, and both had the same results. Presented histograms consist of 1,320 ± 58 (Mean ± SEM) events.

Figure S7: Comparison of CD3 microbead stimulation versus plate-bound CD3/28 mAb stimulation using CD4$^-$ depleted cells as suppressor cells

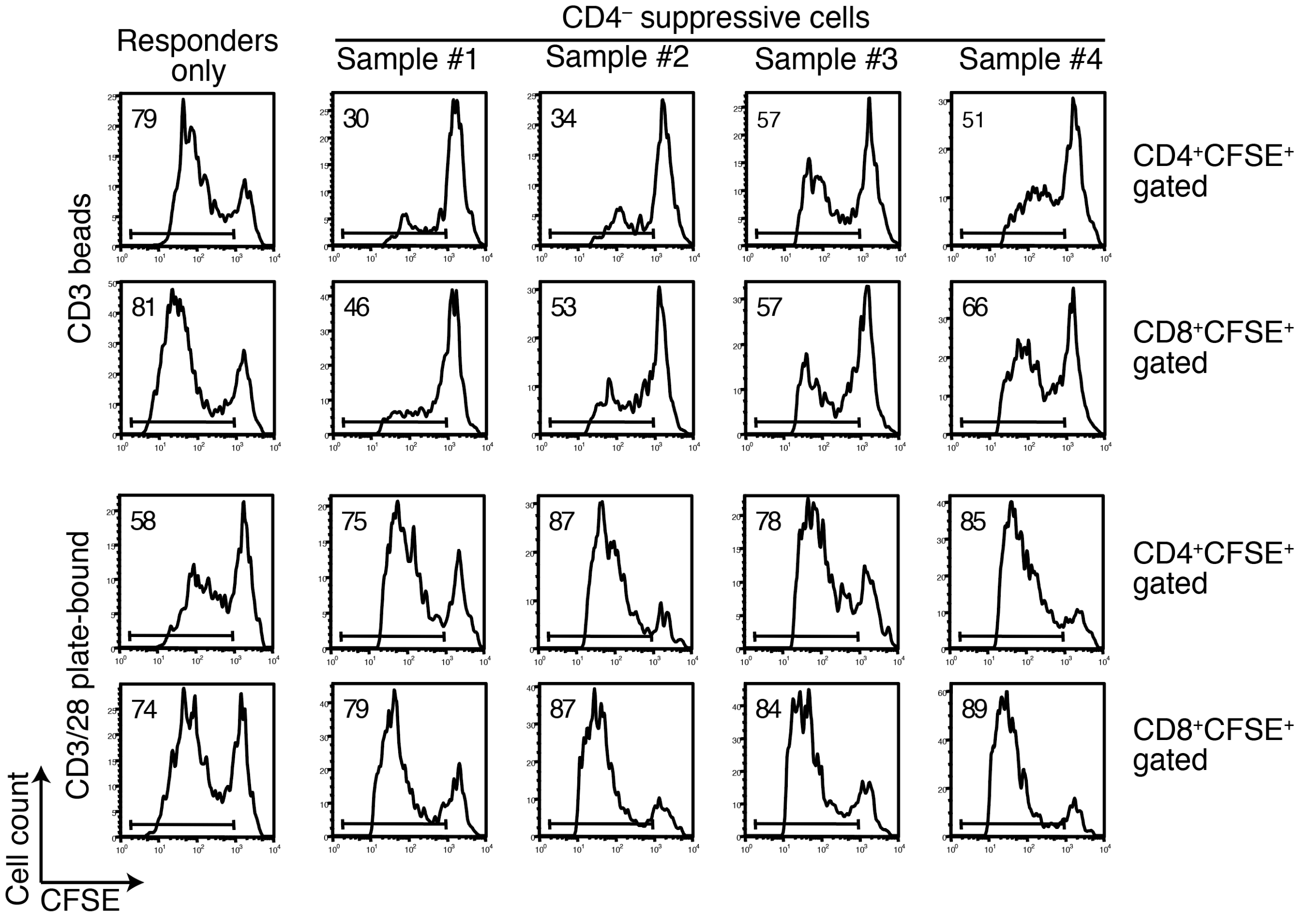

CD4$^-$ depleted cells were isolated using second day blood from PBMC of two anxiety/depression patients (samples #1 and #2), and from tumor (sample #3) and PBMC (sample #4) of a lung cancer patient. Isolated CD4$^-$ cells were incubated with healthy donor #390 responder cells in suppression assay. Responders were stimulated with CD3 microbeads or with plate-bound CD3/28 mAbs. One experiment from two is shown. In total, 7 samples, including healthy donors, were evaluated with the same results. Presented histograms consist of 1,981 ± 149 (Mean ± SEM) events.

Figure S8: Comparison of CD3 microbead stimulation vs. plate-bound CD3 mAb stimulation using $CD4^{-}$ depleted cells as suppressors

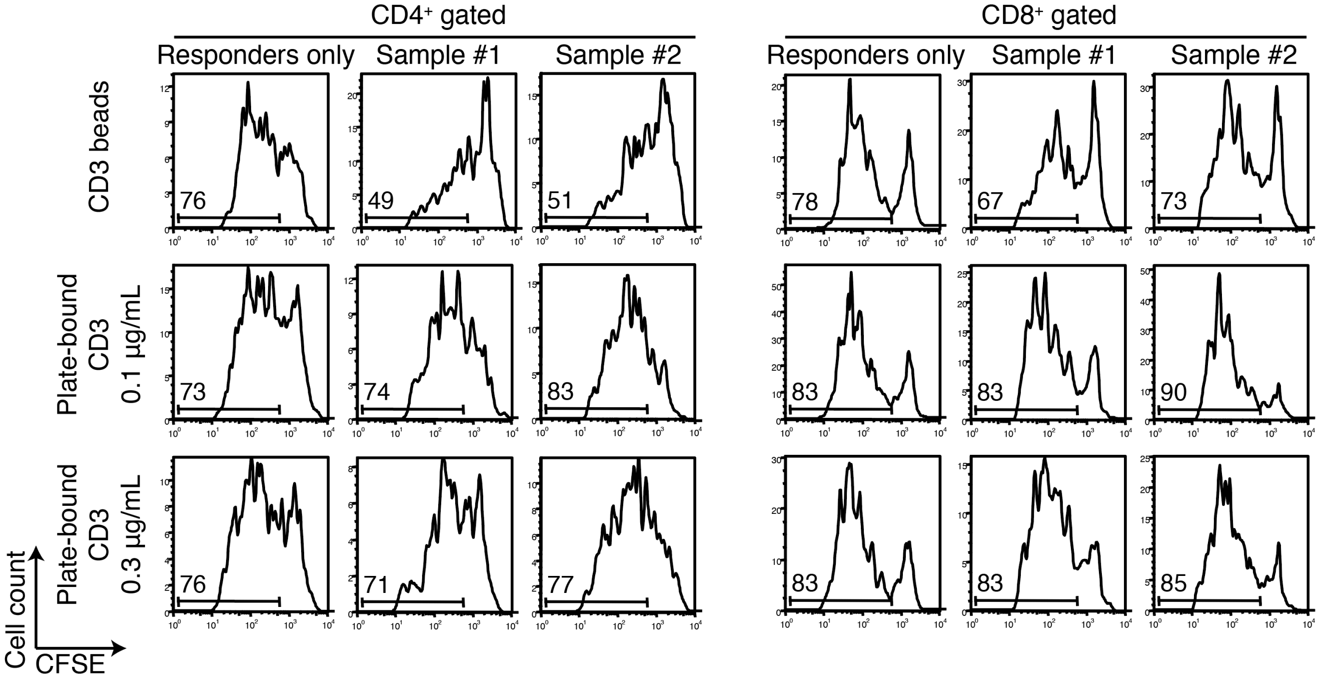

$CD4^{-}$ depleted cells were isolated from the first day PBMC of 2 healthy donors, and incubated with healthy donor #390 responder cells in suppression assay. Responders were stimulated with CD3 microbeads or with plate-bound CD3 mAbs in indicated concentrations. In total, 7 samples, including healthy donors, were evaluated in 3 experiments with the same results. Presented histograms consist of 1,520± 71 (Mean ± SEM) events.

Figure S9: Comparison of CD3 microbead stimulation vs. of CD3/CD28 microbead stimulation using CD4⁻ depleted cells as suppressors

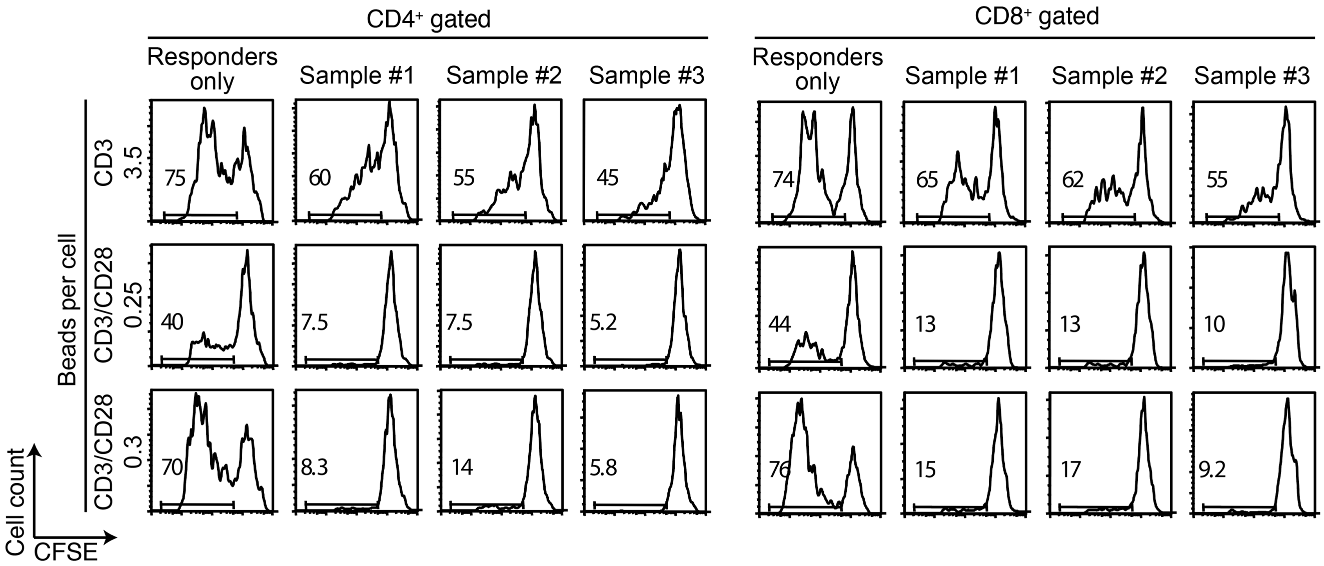

CD4- depleted cells were isolated at first day from PBMC of 2 healthy donors (samples #1 and #2) and one lung transplant patient (sample #3), and incubated with healthy donor ID#390 responder cells in suppression assay. Responders were stimulated with CD3 microbeads or with CD3/28 microbeads in indicated concentrations. In total, 6 samples, including healthy donors, were evaluated in 4 experiments with the same results. Presented histograms consist of 1,818± 70 (Mean ± SEM) events.

Figure S10: Phenotype and suppressive capabilities of neutrophils with different types of T cells stimulation

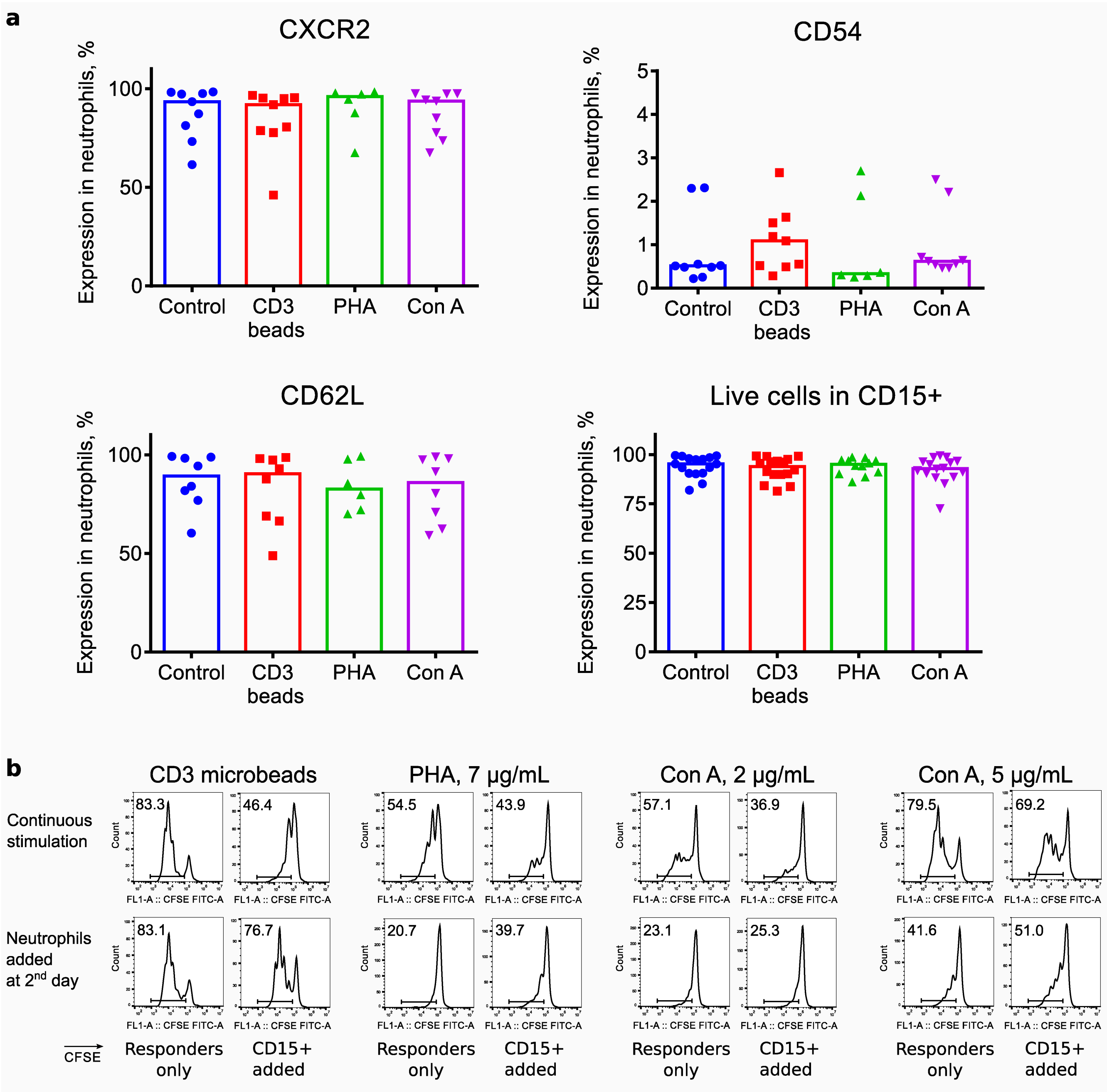

(A) CD15^{+} cells were incubated for 2 hours at 37°C in cell culture media in presence of CD3 microbeads (3.5 beads per cell), Phytohemagglutinin (PHA, 7 μg/mL) or Concanavalin A (Con A, 5 μg/mL), then evaluated with flow cytometry with live/dead staining, CD15 and the following activation markers:

CXCR2, CD54 and CD62L. At least 3 samples from each group, either first day blood or second day blood samples, were evaluated in 5 independent experiments. Medians and individual values are shown. Bars illustrate an absence of apparent activation of neutrophils and the well-preserved viability, although both lectins tend to decrease CD62L expression on neutrophils.

(B) $CD15^+$ cells were isolated with magnetic beads and incubated in 1:1 ratios with CFSE-labeled healthy donor responder PBMC for 4 days. PBMC were stimulated as indicated. As expected, neutrophils suppressed T cell proliferation induced by CD3 microbeads stimulation.Surprisingly, neutrophils also suppressed PHA and Con A - stimulated PBMC in most cases (top row). However, when responder PBMC cells were pre-activated with the same mitogens overnight, and then washed and mixed 1:1 with neutrophils, we observed an absence of any suppression and even, to some extent, an activation of lectins-stimulated PBMC division by neutrophils (bottom row). Importantly that CD3 microbeads were pelleted down along with PBMC, and therefore neutrophils added the next day into suppression assay were still able to disrupt beads-activated divisions of T cells (bottom row, left columns). Divisions of $CD8^+$ cells of one healthy donor in one experiment are shown. Data of $CD4^+$ responders from the same experiment are presented in Figure 6B, and more in depth analysis of T cells proliferation is shown at Suppl. Figure S11). In total, 3 experiments with 4 healthy donors neutrophils and 8 healthy donors PBMC responders were performed with similar results. Presented histograms consist of 3,563 ± 194 (Mean ± SEM) events.

Figure S11: In depth analysis of suppressive capabilities of neutrophils with different types of T cells stimulation

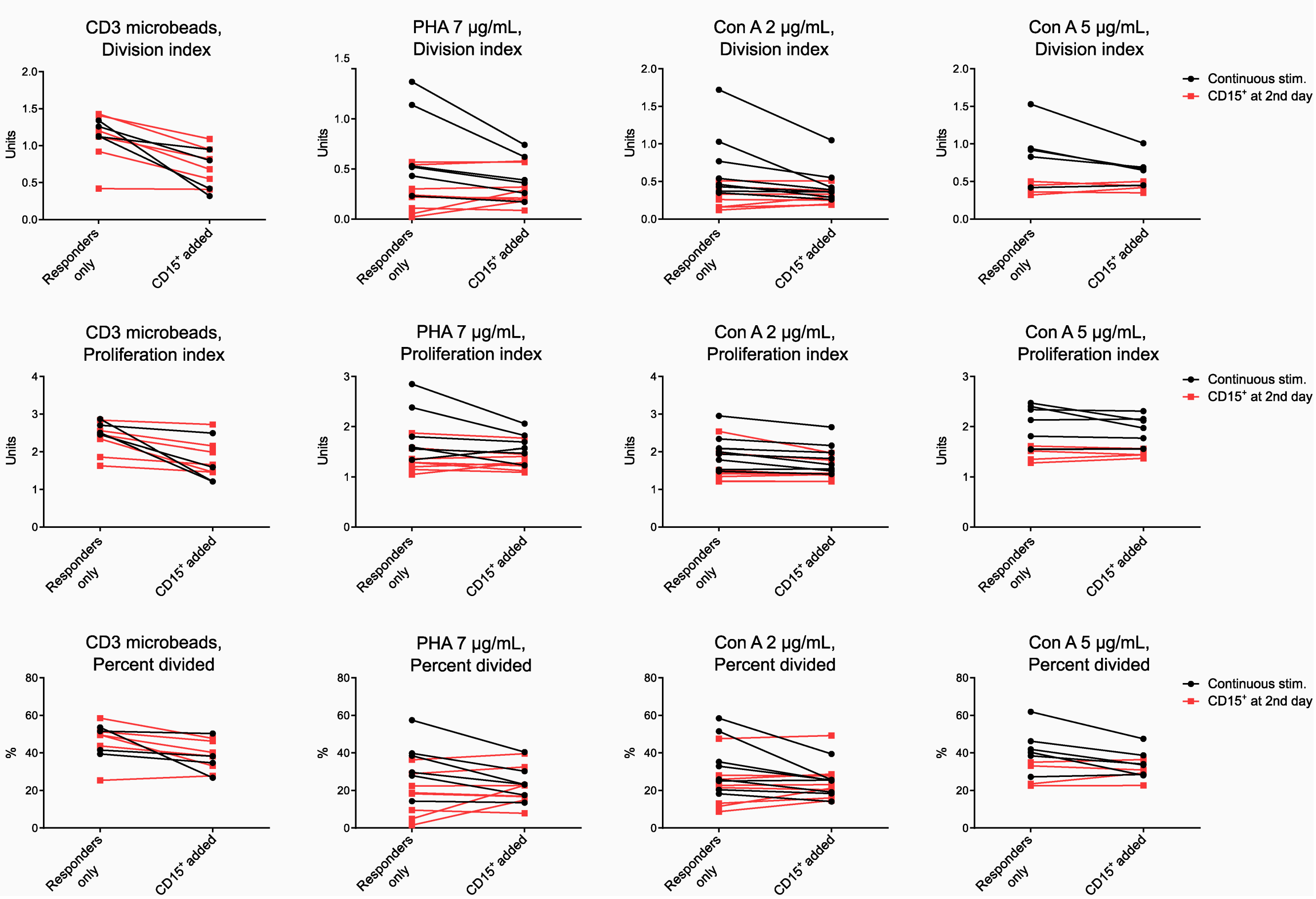

CD15$^+$ cells were isolated with magnetic beads and incubated in 1:1 ratios with CFSE-labeled healthy donor responder PBMC for 4 days. PBMC were stimulated as indicated. CFSE$^+$CD4$^+$ and CFSE$^+$CD8$^+$ responders were gated and analyzed using proliferation modeling in FlowJo v.10.4. Results are presented as Division index (top), Proliferation index (middle) and Percent divided (bottom). As expected, neutrophils suppressed T cell divisions, stimulated with CD3 microbeads. Neutrophils also suppressed PHA and Con A - stimulated PBMC in most cases ("Continuous stimulation", black). However, when responder PBMC cells were pre-activated with the same mitogens overnight, and then washed and mixed 1:1 with neutrophils on the following day, we observed an absence of any suppression for lectins assays ("CD15$^+$ at the 2nd day", red). Data of 1 experiment out of 3 are shown.

S12: Morphological evaluation of neutrophils, incubated with different T cells stimulators. control

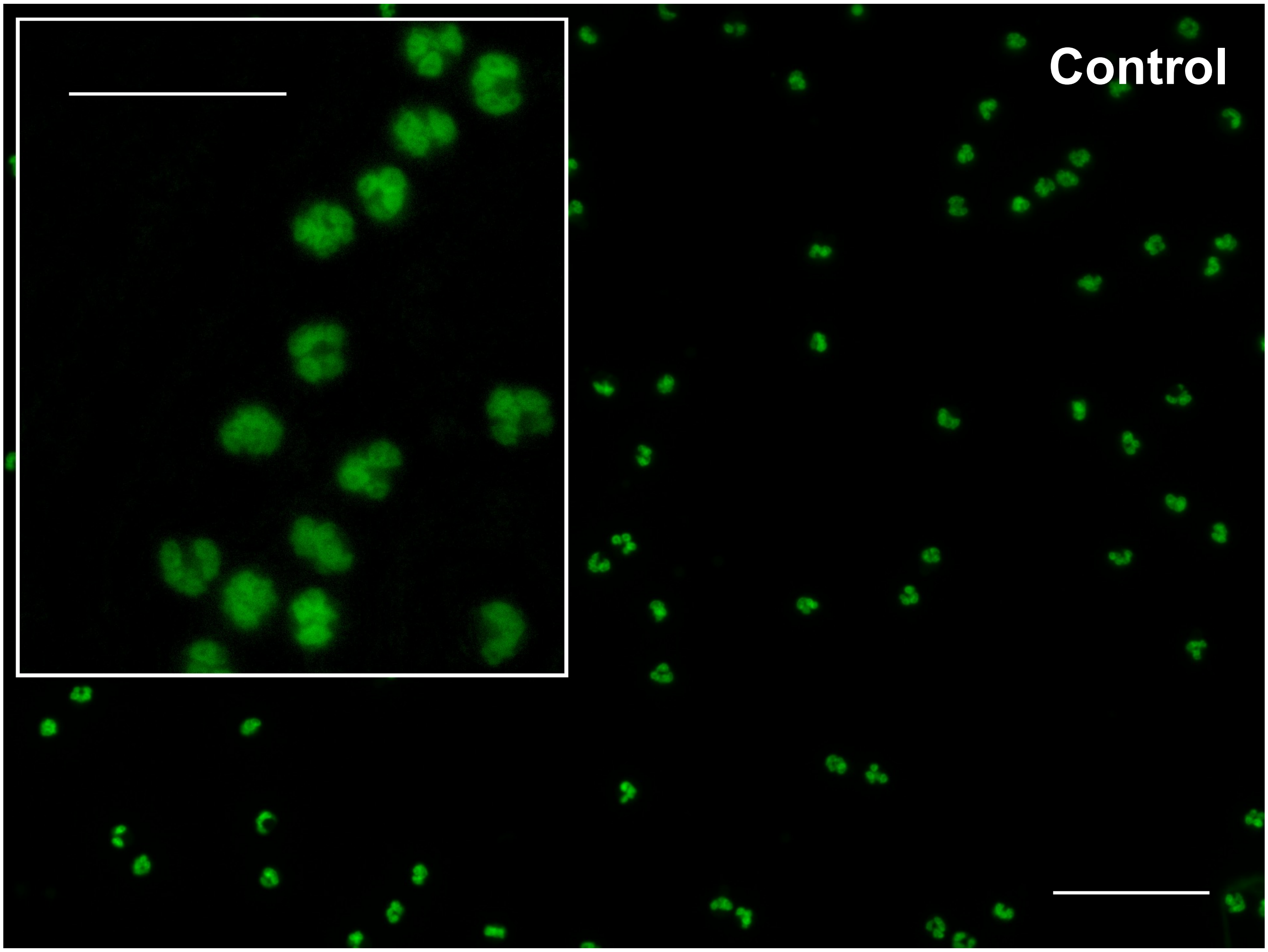

Healthy donors CD15^{+} neutrophils were isolated with magnetic beads, and incubated for 1 hour at 37°C, 5% CO_2 in cell culture media on sterile microscopic slides. Then slides were washed, fixed in 4% formaldehyde and stained with Sytox green (1µM) or DAPI (Mounting Medium with DAPI, not shown). Scales are 50 µm (main image) and 25 µm (in the insert). Neutrophils represent normal appearance with no signs of aggregation and NET production. One experiment out of two is shown.

S13: Morphological evaluation of neutrophils, incubated with different T cells stimulators. CD3 microbeads

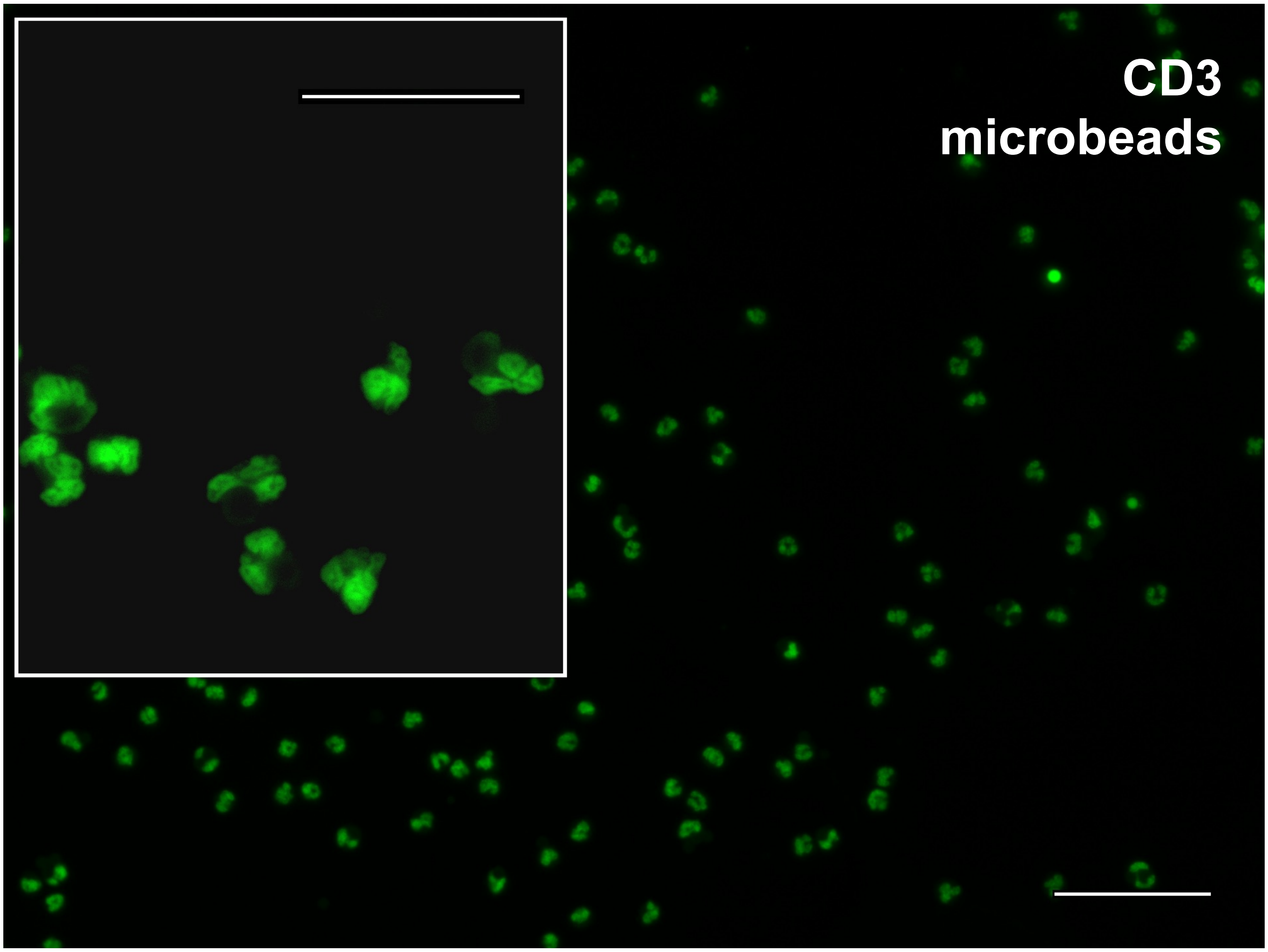

Healthy donors CD15^{+} neutrophils were isolated with magnetic beads, and incubated for 1 hour at 37°C, 5% CO_2 in cell culture media on sterile microscopic slides in presence of CD3 microbeads (3.5 beads per cell). Then slides were washed, fixed in 4% formaldehyde and stained with Sytox green (1µM) or DAPI (Mounting Medium with DAPI, not shown). Scales are 50 µm and 25 µm (in the insert). Neutrophils represent normal appearance and aggregate microbeads, the round shapes of unstained microbeads may be seen on insert. No signs of NET production are seen. One experiment out of two is shown.

S14: Morphological evaluation of neutrophils, incubated with different T cells stimulators.

Phytohemagglutinin (PHA)

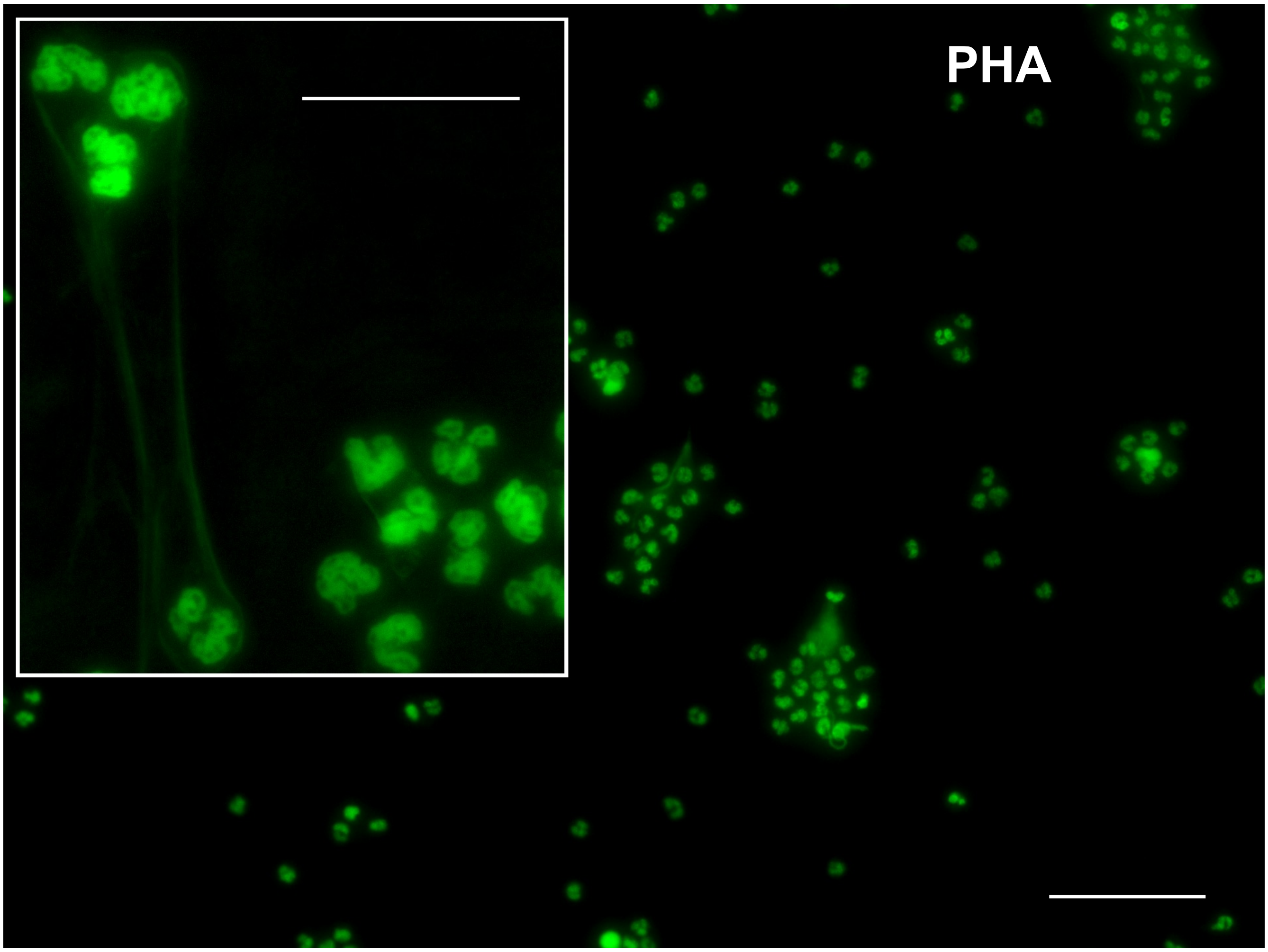

Healthy donors $CD15^+$ neutrophils were isolated with magnetic beads, and incubated for 1 hour at 37°C, 5% CO_2 in cell culture media on sterile microscopic slides in presence of PHA, 7 µg/mL.Then slides were washed, fixed in 4% formaldehyde and stained with Sytox green (1µM) or DAPI (Mounting Medium with DAPI, not shown). Scales are 50 µm and 25 µm (in the insert). Neutrophils represent normal appearance, but form the aggregates and release DNA (NET formation). One experiment out of two is shown.

S15: Morphological evaluation of neutrophils, incubated with different T cells stimulators. Concanavalin A (Con A)

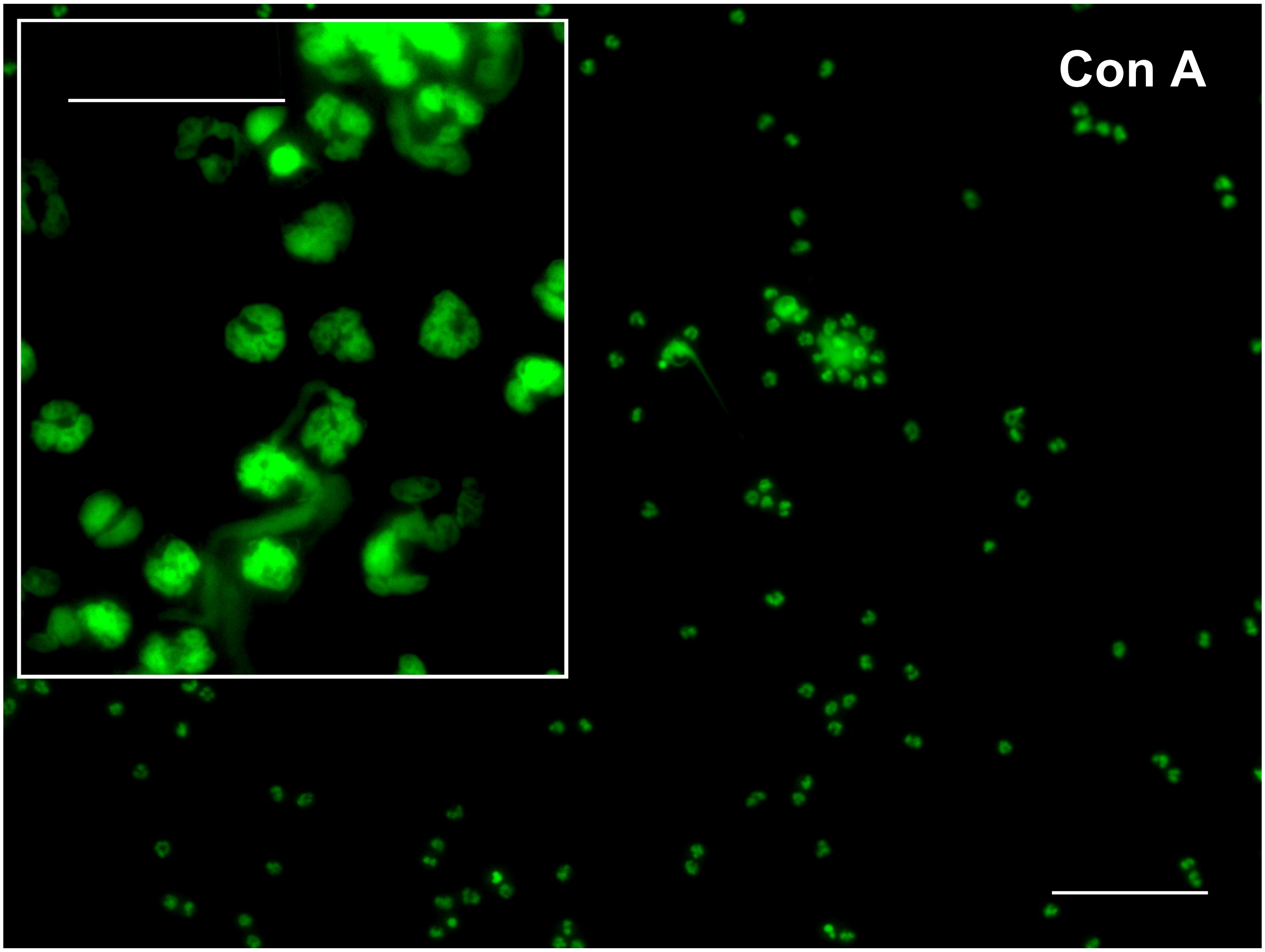

Healthy donors CD15$^+$ neutrophils were isolated with magnetic beads, and incubated for 1 hour at 37°C, 5% CO_2 in cell culture media on sterile microscopic slides in presence of Con A, 5 μg/mL. Then slides were washed, fixed in 4% formaldehyde and stained with Sytox green (1μM) or DAPI (not shown). Scales are 50 μm and 25 μm (an insert). Most neutrophils represent normal appearance, but some of them form the aggregates and release DNA (NET formation). NET-producing cells have more condensed chromatin and demonstrate the disturbed morphological appearance in comparison with PHA-stimulated NET-producing cells. One experiment out of two is shown.

Figure S16: Flow cytometry evaluation of microbeads

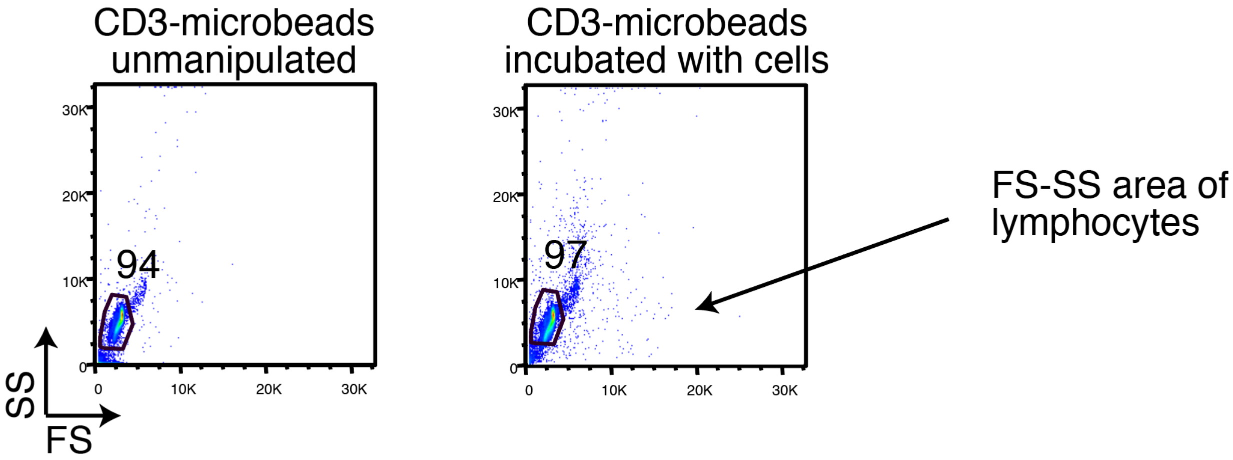

An example of flow cytometry of CD3 mAb-coated microbeads that were dissociated from the cells by vortexing and pipetting, as described in Methods. Microbeads are gated according to their FS-SS properties, and typical lymphocyte FS-SS localization area is empty. In microbeads experiments, 32,313 ± 3,712 (Mean ± SEM) events were evaluated at “microbeads” gates.
